# Circ‐CTNNB1 drives aerobic glycolysis and osteosarcoma progression via m6A modification through interacting with RBM15

**DOI:** 10.1111/cpr.13344

**Published:** 2022-10-01

**Authors:** Feng Yang, Yangyang Liu, Jun Xiao, Bo Li, Yajun Chen, Anpei Hu, Jin Zeng, Zhili Liu, Hucheng Liu

**Affiliations:** ^1^ Department of Orthopedic Surgery The First Affiliated Hospital of Nanchang University Nanchang People's Republic of China; ^2^ Department of Oncology The First Affiliated Hospital of Nanchang University Nanchang People's Republic of China; ^3^ Department of Pathology The First Affiliated Hospital of Nanchang University Nanchang People's Republic of China; ^4^ Department of Pathology, Union Hospital, Tongji Medical College Huazhong University of Science and Technology Wuhan People's Republic of China; ^5^ Department of Pediatric Surgery, Union Hospital, Tongji Medical College Huazhong University of Science and Technology Wuhan People's Republic of China

## Abstract

**Objectives:**

Circular RNAs (circRNAs) are a subclass of noncoding RNAs, playing essential roles in tumorigenesis and aggressiveness. Recent studies have revealed the pivotal functions of circ‐CTNNB1 (a circular RNA derived from CTNNB1) in cancer progression. However, little is known about the role of circ‐CTNNB1 in osteosarcoma (OS), a highly malignant bone tumour in children and adolescents.

**Methods:**

Circ‐CTNNB1 was analysed by qRT‐PCR, and the results were confirmed by Sanger sequencing. The interaction and effects between circ‐CTNNB1 and RNA binding motif protein 15 (RBM15) were analysed through biotin‐labelled RNA pull‐down and mass spectrometry, in vitro binding, and RNA electrophoretic mobility shift assays. In vitro and in vivo experiments were performed to evaluate the biological functions and underlying mechanisms of circ‐CTNNB1 and RBM15 in OS cells.

**Results:**

Circ‐CTNNB1 was highly expressed in OS tissues and predominantly detected in the nucleus of OS cells. Ectopic expression of circ‐CTNNB1 promoted the growth, invasion, and metastasis of OS cells in vitro and in vivo. Mechanistically, circ‐CTNNB1 interacted with RBM15 and subsequently promoted the expression of hexokinase 2 (HK2), glucose‐6‐phosphate isomerase (GPI) and phosphoglycerate kinase 1 (PGK1) through N6‐methyladenosine (m6A) modification to facilitate the glycolysis process and activate OS progression.

**Conclusions:**

Circ‐CTNNB1 drives aerobic glycolysis and OS progression by facilitating RBM15‐mediated m6A modification.

## Graphical abstract

Circ‐CTNNB1 was highly expressed in osteosarcoma (OS) tissues and predominantly detected in the nucleus of OS cells. Ectopic expression of circ‐CTNNB1 promoted the growth, invasion, and metastasis of OS cells in vitro and in vivo. Mechanistically, circ‐CTNNB1 interacted with RBM15 and subsequently promoted the expression of hexokinase 2 (HK2), glucose‐6‐phosphate isomerase (GPI), and phosphoglycerate kinase 1 (PGK1) through N6‐methyladenosine (m6A) modification to facilitate the glycolysis process and activate OS progression. Circ‐CTNNB1 drives aerobic glycolysis and OS progression by facilitating RBM15‐mediated m6A modification.

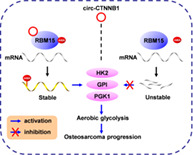

## 1 INTRODUCTION

Osteosarcoma (OS) is the most prevalent type of primary bone cancer and extremely malignant tumour occurs mainly in children and adolescents, with a 5‐year survival rate of less than 30% after the development of metastases.^1^, ^2^, ^3^ With the application of chemotherapy and surgery over the last 30 years, the prognosis of OS remains poor for high rate of metastasis and rapid progression.^4^ Therefore, revealing the molecular mechanisms underlying OS development and progression is critical for developing specific therapies.

CircRNAs, a group of transcripts with closed continuous loops generated from a process called back‐splicing, are described as key regulators of gene expression,^5^, ^6^, ^7^ and dysregulated circRNAs have been identified in almost all types of cancer.^8^ Previous studies have reported various circRNA functions and their underlying mechanisms in cellular physiology including sponging miRNA, binding with proteins, and translating into peptides and proteins, exerting transcriptional or translational regulation.^9^, ^10^, ^11^ Recent studies have shown the vital functions of circRNAs in the control of gene expression via physical interaction with proteins. For example, circ‐Amotl1 promotes tumorigenesis through binding to c‐myc and increasing the retention of nuclear c‐myc in breast cancer.^12^ CircAGO2 binds and facilitates the recruitment of HuR protein in the 3′‐untranslated region of the target gene, and promotes cancer progression.^13^ In a previous study, we have identified that circ‐CTNNB1 interacts with protein and drives tumour progression through the activation of the Wnt/β‐catenin signalling pathway in gastric cancer.^14^ However, the specific functions and underlying mechanistic involvement of circ‐CTNNB1 in OS have not yet been explored.

N6‐methyladenosine (m6A), which results from the methylation of adenosine at the N6 position of almost every type of RNA molecule, has been reported to play vital roles in cancer biology through modulating RNA maturation, localization, translation and metabolism.^15^, ^16^, ^17^ Aberrant level of global m6A abundance has been reported recently in cancers, and the dysregulation could be associated with malignant progression and clinical outcome.^18^ The biological effects of m6A are mainly regulated by three kinds of methylation modulators, namely methylation transferases (Writers), demethylases (Erasers) and methylated readers (Readers), based on which the m6A modification is involved in regulating gene expression of cancer.^19^, ^20^ The ‘writers’ are proteins involved in catalysing the m6A modification of adenosine on target RNA. Accumulating evidence has demonstrated that some methylation modulators are regulated by circRNAs. For example, circKIAA1429 maintains the expression of Zeb1 in a YTHDF3 (a m6A reader protein)‐dependent manner to accelerate the progression of liver cancer,^21^ circMAP2K4 promoted hepatocellular carcinoma cell proliferation by expediting YTHDF1 expression through m6A RNA methylation.^22^

In this study, we detected high expression of circ‐CTNNB1 in OS tissues and cell lines, which was associated with lung metastasis in OS patients and malignant progression of OS cells in vitro and in vivo. Mechanistically, circ‐CTNNB1 interacts with m6A regulator RBM15, which facilitates the ability of the latter to elevate the m6A levels at the 3′‐UTR of the key aerobic glycolysis genes hexokinase 2 (HK2), glucose‐6‐phosphate isomerase (GPI) and phosphoglycerate kinase 1 (PGK1), ensuring a more stable activity and elevated expression of the target genes. Therefore, OS cells are able to obtain metabolic survival advantage from aerobic glycolysis via the upregulation of circ‐CTNNB1.

## 2 MATERIALS AND METHODS

### 2.1 Clinical samples and cell culture

Human samples of OS tissues (*n* = 20) were collected from patients who underwent surgery at The First Affiliated Hospital of Nanchang University, and patients received no preoperative treatment prior to the sample collection. The adjacent non‐tumour tissues were collected as control samples. All procedures were approved by the Ethics Committee of The First Affiliated Hospital of Nanchang University and carried out in accordance with the Helsinki Principles. Written informed consent was provided by all patients. Fresh human samples validated by pathological diagnosis were frozen in liquid nitrogen and stored at −80°C until RNA extraction. The human OS cell lines 143B, HOS, MG‐63, SJSA‐1, Saos‐2 and U2OS and non‐tumour control cell line hFOB 1.19 were cultured in DMEM supplemented with 10% foetal bovine serum (FBS).

### 2.2 Real‐time PCR and quantitative real‐time PCR

The detection of circRNA, mRNA samples were described before,^14^ and primers are shown in Table S1.

### 2.3 Northern blot

The junction probe for circ‐CTNNB1 was synthesized and labelled with digoxigenin, as described in our previous study.^14^

### 2.4 Western blot

Western blot analysis was performed as described before with antibodies specific for β‐actin (ab125402), β‐catenin (ab32572), RBM15 (ab244374), ENO1 (ab155102), GPI (ab66340), PGK1 (ab38007), Flag (ab45766, Abcam, USA), ALDOA (sc‐390733) and IGF2BP1 (sc‐166344, Santa Cruz Biotechnology, USA).

### 2.5 Plasmid construction and stable transfection

Human RBM15 cDNA (2934 bp) was synthesized by TsingKe Biotech Company (Wuhan, China), and the truncations of RBM15 were obtained by PCR amplification with differential primer pairs (Table S2) and subcloned into pCMV‐3Tag‐1A (Addgene, Cambridge, USA). Oligonucleotides specific for shRNAs against circ‐CTNNB1 or RBM15 (Table S2) were inserted into GV298 (Genechem Co., Ltd., Shanghai, China). Stable cell lines were obtained, followed by selection with neomycin or puromycin (Invitrogen) for 2–3 weeks.

### 2.6 RNA fluorescence in situ hybridization (RNA‐FISH)

A biotin‐labelled antisense probe for the circ‐CTNNB1 junction sequence and probes for GAPDH and U1 were synthesized as we previously described.^14^ The probes were hybridized using the Fluorescent In Situ Hybridization kit (RiboBio) following the manufacturer's instructions. The nuclei of OS cells were counterstained with 4′,6‐diamidino‐2‐phenylindole (DAPI), and the images were analysed using a Nikon A1Si Laser Scanning Confocal Microscope (Nikon, Japan).

### 2.7 Dual‐luciferase reporter assay

The TOP‐FLASH and FOP‐FLASH reporters for the activity of the canonical Wnt pathway were obtained from Millipore (Temecula, CA, USA). The promoter fragments of human HK2 (−1813/+424), GPI (−1854/+247), PGK1 (−882/+246) and 3′‐UTR of target genes amplified from genomic DNA (Table S2) were subcloned into pGL3‐basic and psiCHECK2. Mutations of m6A sites at the 3′‐UTR were performed with the GeneTailor™ Site‐Directed Mutagenesis System (Invitrogen). The dual‐luciferase assay was performed according to the manufacturer's instructions (Promega). The luciferase signal in the promoter activity assay was normalized to the firefly/Renilla ratio, while the activity of the 3′‐UTR reporter was measured by the Renilla/firefly ratio.

### 2.8 Biotin‐labelled RNA pull down and mass spectrometry analysis

The biotin‐labelled RNA probe for circ‐CTNNB1 was in vitro transcribed using the Biotin RNA Labeling Mix kit (Roche) and T7 RNA polymerase, as described in our previous study.^23^ RNA pull‐down assay was performed at room temperature, and the biotinylated proteins were detected by mass spectrometry (MS) at the Wuhan Institute of Biotechnology (Wuhan, China).

### 2.9 Fluorescence immunocytochemical staining

OS cells were grown on coverslips, incubated with 5% milk for 1 h, and treated with an antibody specific for RBM15 (ab244374, Abcam, USA) at 4°C overnight. Then, the cells were stained with Alexa Fluor 594 IgG and DAPI. The images were photographed under a Nikon A1Si Laser Scanning Confocal Microscope (Niko, Japan).

### 2.10 Aerobic glycolysis and seahorse extracellular flux assays

Cellular Aerobic glycolysis activity and glucose uptake, lactate production and adenosine triphosphate (ATP) levels were detected as previously described.^23^ Extracellular acidification rate and oxygen consumption rate (ECAR, OCR) were measured in response to glucose (10 mM), oligomycin (2 uM) and 2‐deoxyglucose (2‐DG, 100 mM) under basal conditions with a Seahorse Biosciences XFe24 Flux Analyzer (North Billerica, MA, USA).

### 2.11 Cross‐linking RIP assay

Cells were ultraviolet light cross‐linked at 254 nm (200 J/cm^2^) in PBS and collected by scraping, and RNA immunoprecipitation (RIP) assay was performed according to the instructions of Magna RIPTM Kit (Millipore), with antibodies specific for RBM15 (ab244374, Abcam, USA) and IGF2BP1 (sc‐166344, Santa Cruz Biotechnology, USA). Co‐precipitated RNAs were detected by RT‐PCR or real‐time quantitative PCR with specific primers (Table S1).

### 2.12 In vitro binding assay

Five truncates of RBM15 were cloned with primers (Table S1) into vectors with flag tags as we described previously.^13^ The Flag‐RBM15 and circ‐CTNNB1 complexes were pulled down using Flag beads (Sigma, USA). Circ‐CTNNB1 was measured by RT‐PCR with divergent primers (Table S1), and protein was validated by western blot.

### 2.13 RNA electrophoretic mobility shift assay (EMSA)

Biotin‐labelled circ‐CTNNB1 probe was prepared as described above. RNA EMSA was conducted according to the instructions of LightShift Chemiluminescent RNA EMSA Kit (Thermo Fisher Scientific, Inc.)

### 2.14 In vitro cell viability, growth, and invasion assays

The in vitro viability, growth and invasion capabilities of OS cells were detected by MTT colorimetry, colony formation and matrigel invasion assays, as described previously.^24^

### 2.15 Xenografts in mice

For in vivo tumour growth studies, 143B cells were subcutaneously injected into the dorsal flanks of 5‐week‐old male BALB/c nude mice (*n* = 5 per group) in a blind, randomized fashion. The growth and weight of xenografts were detected 1 month later. In experimental metastasis studies, tail vein injection of 143B cells was performed in a blind, randomized fashion in 5‐week‐old male BALB/c nude mice (*n* = 5 per group). Metastasis counts and survival time of each mouse were monitored and recorded, and the xenografts were studied by haematoxylin and eosin (H&E) staining. All animal experiments were performed in accordance with the NIH Guidelines for the Care and Use of Laboratory Animals and approved by the Animal Care Committee of Tongji Medical College.

#### 2.15.1 Immunohistochemistry

Antibodies against Ki‐67 (ab92742), CD31 (ab28364) were used for IHC as reported previously.^25^

### 2.16 Statistical analysis

All data are presented as the mean ± standard error of the mean (SEM) processed by GraphPad Prism 5.0 (La Jolla, USA). Student's *t*‐test or one‐way analysis of variance (one‐way ANOVA) was used to evaluate differences between groups. All statistical tests were two sided. A value of *p* < 0.05 was considered statistically significant.

## 3 RESULTS

### 3.1 Circ‐CTNNB1 is upregulated in human OS tissues and cells

**FIGURE 1:**
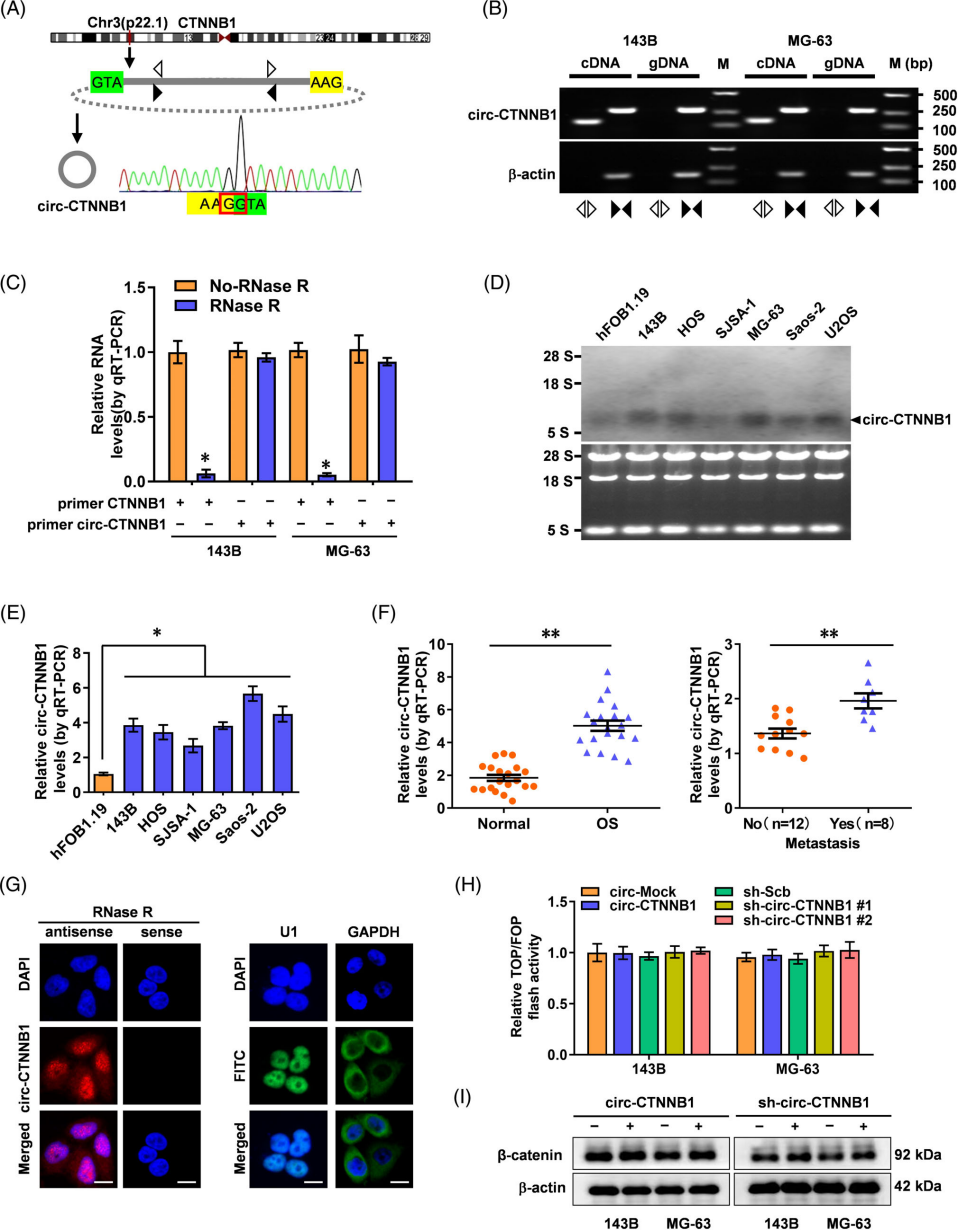
Expression of circ‐CTNNB1 in OS cell lines and tissues, and subcellular location of circ‐CTNNB1. (A) Schematic illustration of the genomic location of circ‐CTNNB1 derived from its host gene and validation by Sanger sequencing. (B) RT‐PCR assay showing the presence of circ‐CTNNB1 with divergent and convergent primers from cDNA or genomic DNA (gDNA) of differential OS cell lines using β‐actin as the negative control. (C) qRT‐PCR analysis of the expression of circ‐CTNNB1 after RNase R treatment in 143B or MG‐63 cells. (D) Northern blot showing the differential expression of circ‐CTNNB1 in hFOB 1.19 cells and OS cell lines. (E, F) qRT‐PCR assay showing the relative levels of circ‐CTNNB1 (normalized to β‐actin) in cultured cell lines and human tissues. (G) RNA‐FISH assay showing the nuclear localization of circ‐CTNNB1. Scale bar: 10 μm. (H, I) TOP/FOP flash assay (H) and Western blot (I) assay revealing the β‐catenin activity and protein levels of CTNNB1 in 143B and MG‐63 cells. (Data were mean ± SEM of three experiments. Student's *t*‐test and ANOVA compared the difference in C, E, F, H. *p < 0.05, **p < 0.01)

In a previous study, we identified that circ‐CTNNB1 drives cancer growth, invasion, and metastasis through the activation of β‐catenin in cancer,^14^ while no further studies have been conducted on this circRNA in OS. The generation of circ‐CTNNB1 from CTNNB1 was analysed by RT‐PCR with divergent primers, and Sanger sequencing confirmed the predicted back‐splicing junction in OS cells (Figure 1A). Furthermore, using divergent primers of circ‐CTNNB1, PCR products could only be amplified from cDNA but not from genomic DNA in 143B and MG‐63 cells (Figure 1B). Circ‐CTNNB1 was resistant to RNase R digestion, while the linear RNA of CTNNB1 was significantly reduced after RNase R treatment in 143B and MG‐63 cells (Figure 1C). Higher endogenous expression levels of circ‐CTNNB1 were observed in OS cells than those of hFOB 1.19 cells in qRT‐PCR assay (Figure 1D) and Northern blot assay (Figure 1E). Moreover, we detected the expression profiles in samples of clinical patients with OS tissues, revealing that circ‐CTNNB1 was upregulated and associated with lung metastasis in OS patients (Figure 1F). Subcellular fractionation and RNA fluorescence in situ hybridization (FISH) assays indicated the nuclear enrichment of circ‐CTNNB1 in OS cells (Figure 1G; Figure S1A). To investigate the cis‐acting properties of circ‐CTNNB1 in regulating β‐catenin signalling, overexpression or knockdown of circ‐CTNNB1 in OS cells was established (Figure S1B,C). The TOP/FOP flash assay revealed that neither ectopic expression nor knockdown of circ‐CTNNB1 affected the RNA transcripts, protein expression, or β‐catenin activity in OS cells (Figure S1C, Figure 1H,I). These results demonstrated a relatively higher expression of circ‐CTNNB1 in OS tissues and cells.

### 3.2 Circ‐CTNNB1 exerts an oncogenic role in OS progression in vitro and in vivo

**FIGURE 2:**
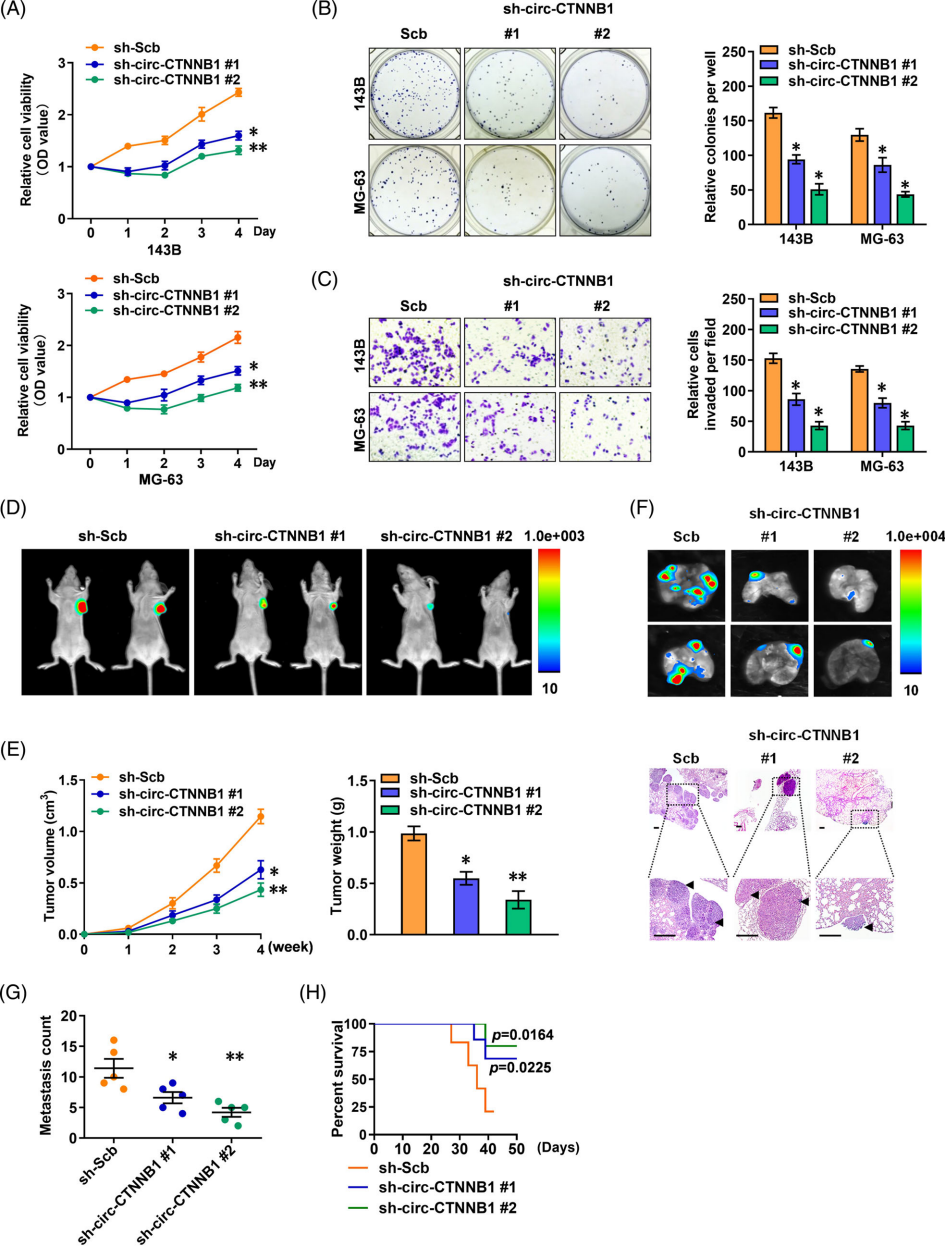
Knockdown of circ‐CTNNB1 inhibits the growth and aggressiveness of OS in vitro and in vivo. (A) MTT assay of 143B and MG‐63 cells stably transfected with scrambled shRNA (sh‐Scb) and sh‐circ‐CTNNB1#1, #2. (B, C) In vitro growth and invasion of 143B and MG‐63 cells stably transfected with sh‐Scb and sh‐circCTNNB1#1, #2, as revealed by colony formation (B) and matrigel invasion (C) assays. (D, E) 143B cells with stable expression of sh‐Scb and sh‐circ‐CTNNB1#1, #2 were injected into the dorsal flanks of nude mice (*n* = 5 for each group). In vivo growth curve and weight measured at the end point of the xenografts. (F–H) 143B cells stably transfected with sh‐Scb and sh‐circ‐CTNNB1#1, #2 were injected into the tail vein of nude mice (*n* = 5 for each group). Representative images of HE staining (F, scale bar: 50 μm, the upper magnification is ×40 and the lower magnification is ×200), quantification of lung metastatic colonization (G), and collected Kaplan–Meier curves (H). (Data were mean ± SEM of three experiments. ANOVA analysed the difference in A–C, E, G. Log‐rank test for survival comparison in H. *p < 0.05 vs. sh‐Scb, **p < 0.01 vs. sh‐Scb) [Correction added on 20 December 2022, after first online publication: the images of HE staining for “sh‐circ‐CTNNB1” group in Fig. 2F has been corrected]

To explore the roles of circ‐CTNNB1 in OS progression, the impacts on the tumorigenesis and aggressiveness were investigated in 143B and MG‐63 cells with stable transfection of two independent shRNAs against circ‐CTNNB1. In MTT colorimetric assay, stable knockdown of circ‐CTNNB1 decreased the vitality of OS cells (Figure 2A). In colony formation and matrigel invasion assays, stable knockdown of circ‐CTNNB1 reduced the growth and invasion capability of 143B and MG‐63 cells (Figure 2B,C). Consistently, stable transfection of sh‐circ‐CTNNB1 resulted in a significant inhibition in the tumour growth of xenografts formed by subcutaneous injection of 143B cells into athymic nude mice (Figure 2D,E). Importantly, in the experimental metastasis assay, nude mice treated with tail vein injection of 143B cells with stable knockdown of circ‐CTNNB1 displayed statistically less lung metastatic colonies and a greater survival probability than the control group (Figure 2F–H). Taken together, these results showed that silencing circ‐CTNNB1 inhibits the growth and aggressiveness of OS cells in vitro and in vivo.

### 3.3 Circ‐CTNNB1 directly interacts with m6A regulator RBM15

**FIGURE 3:**
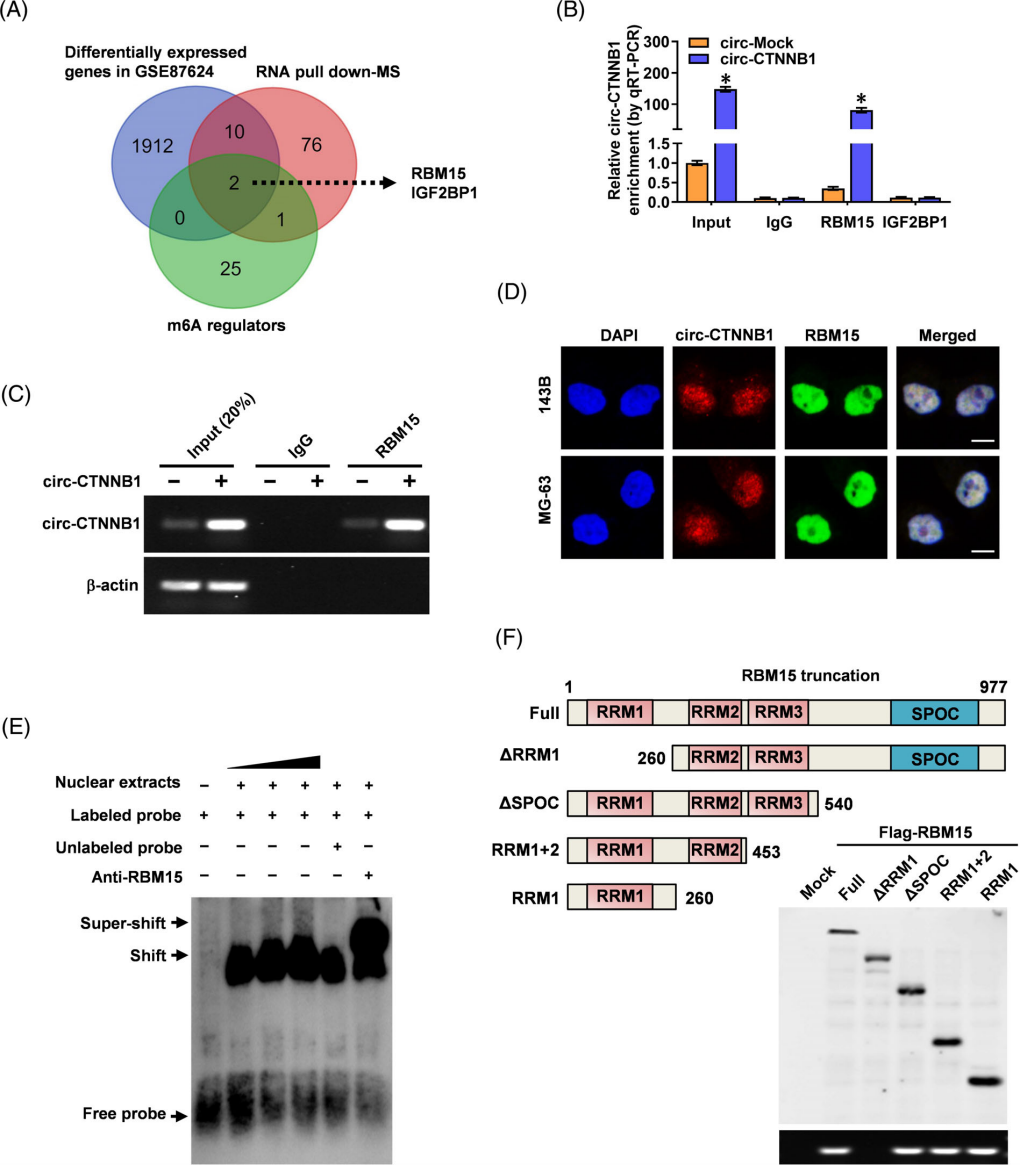
Screening of protein interactions with circ‐CTNNB1. (A) Overlapping analysis (Venn diagram) revealing the m6A regulators that were pulled down by biotin‐labelled circ‐CTNNB1 from the lysates of 143B cells in mass spectrometry (MS) assays with differentially expressed genes in OS cells (GSE87624) and m6A regulators. (B) RIP and qRT‐PCR assays showing the interaction between circ‐CTNNB1 and two proteins in OS cells. (C) RIP assays revealing the interaction between circ‐CTNNB1 and RBM15 in 143B cells stably transfected with circ‐Mock or circ‐CTNNB1. (D) Dual RNA FISH and immunofluorescence staining assays showing the colocalization of circ‐CTNNB1 (red) and RBM15 (green) in cultured 143B and MG‐63 cells with DAPI nuclei staining (blue). Scale bar: 10 μm. (E) RNA EMSA showing the interaction between endogenous RBM15 and biotin‐labelled RNA probe for circ‐CTNNB1 (arrowheads), with RBM15 antibody or competition using an excess of unlabeled homologous circ‐CTNNB1 probe. (F) Schematic diagram showing the domains of RBM15 truncations (upper panel), and in vitro binding assay (lower panel) showing the enriched circCTNNB1 levels detected by RT‐PCR after incubation with full‐length or truncated forms of FLAG‐tagged recombinant RBM15 protein validated by Western blot. (Data were mean ± SEM of three experiments. Fisher's exact test for over‐lapping analysis in A, ANOVA analysed the difference in b. *p < 0.05 vs. IgG)

Previous studies have shown that circRNA can regulate cancer progression through binding with RNA binding proteins. Given the nuclear location of circ‐CTNNB1, we hypothesized that circ‐CTNNB1 may regulate OS progression via potential protein partners. To evidence our hypothesis, we performed a proteomic analysis of circ‐CTNNB1‐associated proteins in 143B cells by the RNA pull‐down assay with a biotin‐labelled probe. m6A is one of the most common and abundant regulation ways of mRNA stability and can further mediate circRNAs function.^20^, ^26^ The MS assay revealed 89 proteins of circ‐CTNNB1 pull‐down, and overlapped it with m6A regulators and differentially expressed genes in GSE87624 to search critical gene aberrantly expressed in OS that binding with circ‐CTNNB1, indicating two potential protein (Figure 3A, Table S3). Furthermore, RIP assay validated the interaction of circ‐CTNNB1 with the m6A writer RNA binding motif protein 15 (RBM15) but not with insulin‐like growth factor 2 mRNA‐binding protein 1 (IGF2BP1) in OS cells (Figure 3B). Notably, the expression levels of RBM15 were upregulated in OS (Figure S3A–C), and circ‐CTNNB1 has no regulatory effect on RBM15 in OS cells (Figure S3D,E). Moreover, transfection of circ‐CTNNB1 increased its enrichment in RNA co‐precipitated by RBM15 antibody in 143B cells (Figure 3C). Dual RNA‐FISH and immunofluorescence assays confirmed the nuclear colocalization of circ‐CTNNB1 and RBM15 in 143B and MG‐63 cells (Figure 3D). Consistently, the RNA electrophoretic mobility shift assay (EMSA) showed that circ‐CTNNB1 interacted strongly with endogenous RBM15 in nuclear extracts (Figure 3E). We further investigated the interaction domain between circ‐CTNNB1 and RBM15. The in vitro binding assay indicated that the RRM1 domain, but not other domains of FLAG‐tagged RBM15 protein, was crucial for the interaction of RBM15 with circ‐CTNNB1 (Figure 3F). These results indicated that circ‐CTNNB1 interacted with RBM15.

### 3.4 Circ‐CTNNB1 promotes aerobic glycolysis in OS

**FIGURE 4:**
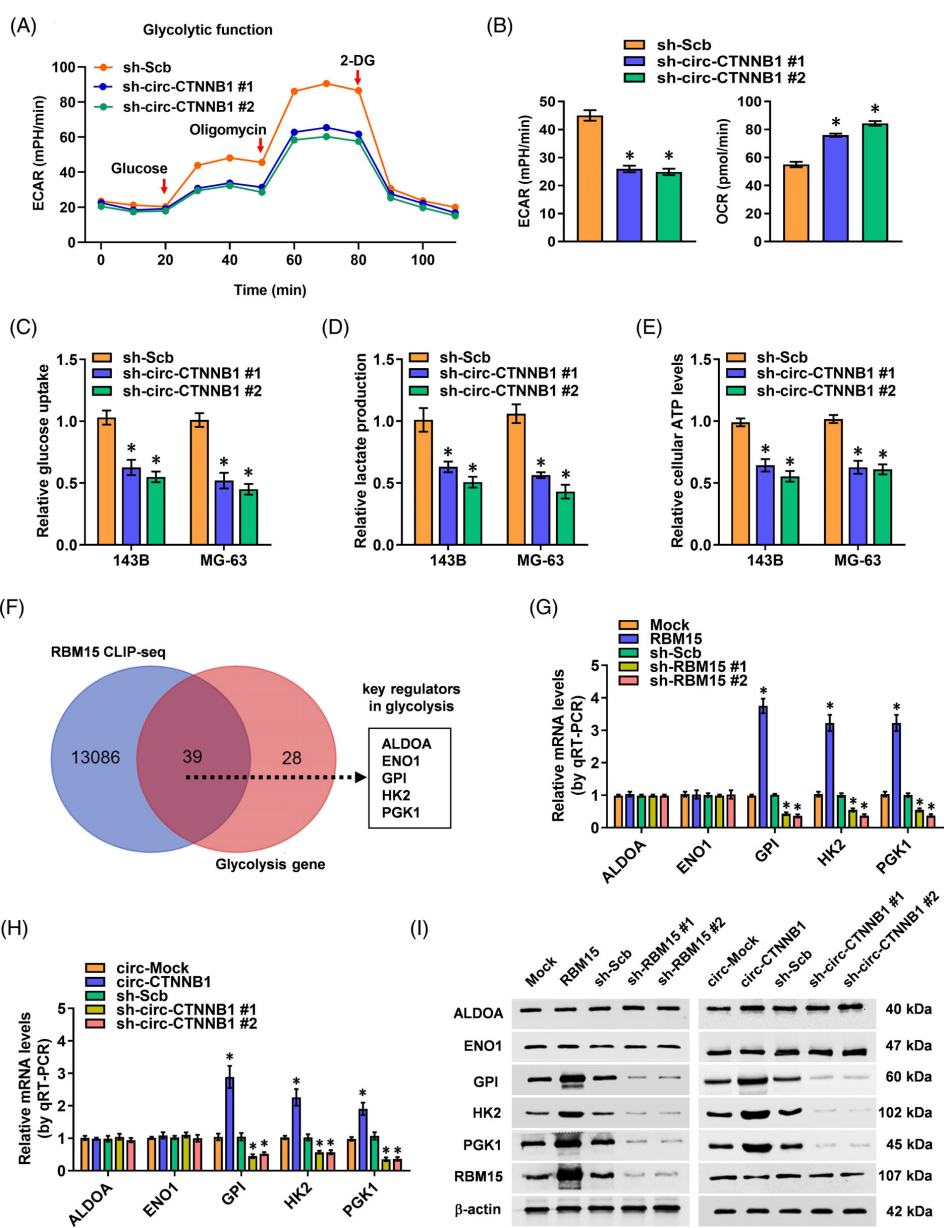
Circ‐CTNNB1 promotes aerobic glycolysis in OS. (A, B) Seahorse tracing curves (A) and ECAR and OCR (B) of 143B cells stably transfected with sh‐Scb and sh‐circ‐CTNNB1#1, #2 or treated with glucose (10 mM), oligomycin (2 μM), or 2‐deoxyglucose (2‐DG, 50 mM) at indicated points. (C–E) Glucose uptake (C), lactate production (D), and ATP levels (E) in 143B and MG‐63 cells stably transfected with sh‐Scb and sh‐circCTNNB1#1, #2. (F) Venn diagram overlapping RBM15 CLIP‐seq with glycolytic genes revealing five target genes involved in the aerobic glycolysis of the circ‐CTNNB1/RBM15 axis. (G–I) Transcript and protein expression levels of ALDOA, ENO1, GPI, HK2, and PGK1 in 143B cells with overexpression or knockdown of RBM15 and circ‐CTNNB1 measured by qRT‐PCR (G, H) and western blot (I) assay. (Data were mean ± SEM of three experiments. Fisher's exact test for over‐lapping analysis in F, Student's *t*‐test and ANOVA analysed the difference in A–E, G, H. *p < 0.05 vs. sh‐Scb, mock or circ‐Mock)

Aerobic glycolysis is a hallmark of metabolic reprogramming in various cancers. However, the mechanisms regulating the glycolytic activity remain elusive in OS. It was reported that circRNA could regulate aerobic glycolysis and cancer progression in an m6A‐dependent manner.^27^, ^28^ Herein, aerobic glycolysis and seahorse extracellular flux assays were conducted to explore the roles of circ‐CTNNB1 in OS. Knockdown of circ‐CTNNB1 attenuated the ECAR and increased the OCR in 143B cells, while ectopic expression of circ‐CTNNB1 significantly promoted the glycolytic process (Figure 4A,B; Figure S2A,B). Accordingly, knockdown or overexpression of circ‐CTNNB1 decreased and increased the glucose uptake, lactate production, and ATP levels of OS cells, respectively (Figure 4C–E; Figure S2C–E). Treatment with 2‐deoxyglucose (2‐DG), an established glycolysis inhibitor, abolished the increase induced by circ‐CTNNB1 overexpression in the glucose uptake, lactate production, ATP levels, proliferation and invasiveness of OS cells in vitro and in vivo (Figure S2C–I). Given the nuclear interaction described above, we investigated the potential effects of circ‐CTNNB1 binding with RBM15 in the aerobic glycolysis in OS. RBM15 was shown to promote the glycolytic process in OS (Figure S4A–E). Five crucial glycolytic genes were identified as targets of the circ‐CTNNB1/RBM15 axis by the comprehensive analysis of RBM15 CLIP‐seq and glycolytic genes (Figure 4F, Table S3). Notably, ectopic expression or knockdown of RBM15 and circ‐CTNNB1 increased and decreased, respectively, the transcripts and protein levels of GPI, HK2 and PGK1, but not of fructose‐bisphosphate A (ALDOA) or enolase 1 (ENO1), in OS cells (Figure 4G–I). These findings indicated that circ‐CTNNB1 promotes aerobic glycolysis in OS cells.

### 3.5 Circ‐CTNNB1 facilitates RBM15‐mediated gene activation via m6A regulation

**FIGURE 5:**
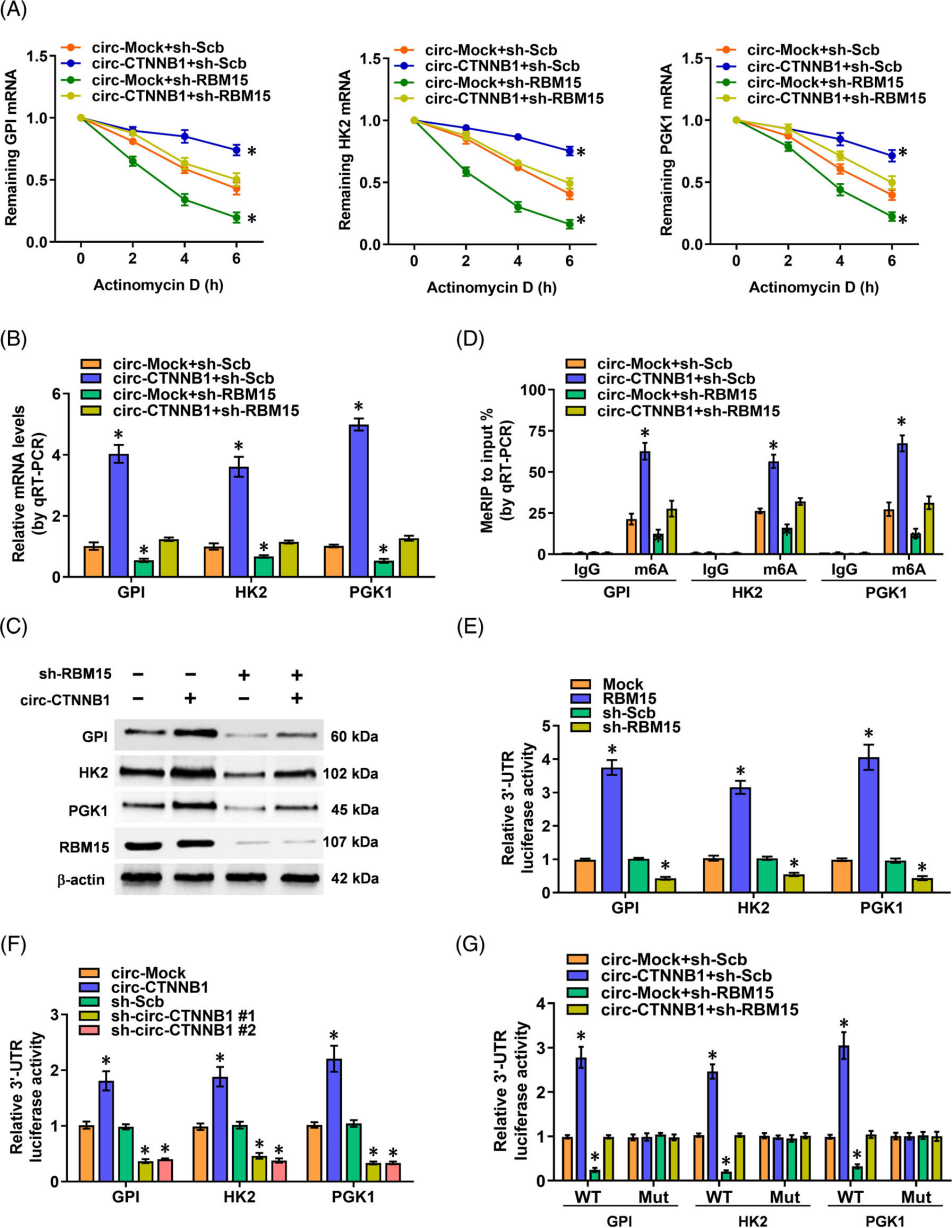
Circ‐CTNNB1 facilitates RBM15‐mediated gene activation via m6A regulation. (A) qRT‐PCR assay indicating the half‐life levels of GPI, HK2, and PGK1 mRNA (normalized to β‐actin) in 143B cells treated with actinomycin D (1 μg/ml) for the indicated periods of time and stably transfected with sh‐Scb or sh‐RBM15 or co‐transfected with circ‐Mock or circ‐CTNNB1. (B, C) qRT‐PCR (B) and western blot (C) assays revealing the transcript and protein expression levels of GPI, HK2, and PGK1 mRNA (normalized to β‐actin) in 143B cells stably transfected with sh‐Scb or sh‐RBM15 or co‐transfected with circ‐Mock or circ‐CTNNB1. (D) MeRIPqPCR assay followed by qRT‐PCR revealing the GPI, HK2, and PGK1 m6A modification in 143B cells stably transfected with sh‐Scb or sh‐RBM15 or co‐transfected with circ‐Mock or circ‐CTNNB1. (E, F) Dual‐luciferase assay revealing the 3′‐UTR activity of GPI, HK2, and PGK1 in MG‐63 cells stably transfected with overexpression or knockdown of RBM15 or circ‐CTNNB1. (G) Wild‐type or m6A site mutation of 3′‐UTR was established, and dual‐luciferase assay revealed the 3′‐UTR activity of GPI, HK2, and PGK1 in OS cells stably transfected with sh‐Scb or sh‐RBM15 or co‐transfected with circ‐Mock or circ‐CTNNB1. (Data were mean ± SEM of three experiments. ANOVA analysed the difference in A, B, D–G. *p < 0.05 vs. circ‐Mock+sh‐Scb, Mock, circ‐Mock, sh‐Scb)

We further investigated the effects of the interplay and the underlying mechanisms between circ‐CTNNB1 and RBM15 on the regulation of target genes (GPI, HK2 and PGK1) and cancer progression in OS cells. As m6A modification is involved in the post‐transcriptional control of gene expression, the mRNA‐stabilizing function was tested. We interfered the process of transcription by the RNA polymerase II inhibitor actinomycin D to observe the degradation rate of mRNA. Notably, the stable ectopic expression of circ‐CTNNB1 abolished the decrease of the half‐life, transcript and protein levels of GPI, HK2 and PGK1 by knockdown of RBM15 in 143B and MG‐63 cells (Figure 5A–C; Figure S5A,B). While, neither ectopic expression nor knockdown of RBM15, could affect the promoter activity of GPI, HK2 and PGK1 in 143B and MG‐63 cells in the dual‐luciferase assay (Figure S5C,D). Next, the RNA methylation quantification assay was used to analyse whether circ‐CTNNB1 and RBM15 regulate target gene expression in a m6A‐dependent manner. As expected, the MeRIP‐qPCR assay showed that the 3′‐UTR of GPI, HK2 and PGK1 was effectively enriched by m6A‐specific antibody, and m6A level was remarkably increased in circ‐CTNNB1‐overexpression cells, which were prevented by knockdown of RBM15 (Figure 5D). Importantly, RBM15 wild type, but not ΔRRM1 truncation of RBM15, abolished the decrease of mRNA half‐life, transcript and m6A levels of GPI, HK2 and PGK1 by knockdown of circ‐CTNNB1 in 143B cells (Figure S5E–G). Therefore, we supposed that circ‐CTNNB1 could regulate the m6A levels of GPI, HK2 and PGK1. The m6A modification was at the 3′‐UTR of the target gene, and several m6A sites were predicted with high confidence by SRAMP (http://www.cuilab.cn/sramp). In the dual‐luciferase assay with a reporter containing the 3′‐UTR of GPI, HK2 and PGK1, ectopic expression or interference of circ‐CTNNB1 and RBM15 promoted and attenuated the 3′‐UTR activity in OS cells, respectively (Figure 5E,F). Importantly, stable overexpression of circ‐CTNNB1 increased the wild‐type 3′‐UTR activity, which was reduced by stable knockdown of RBM15, while the luciferase activity of mutated 3′‐UTR was not affected in OS cells (Figure 5G). These results showed that circ‐CTNNB1 facilitated RBM5‐mediated GPI, HK2, and PGK1 activation via m6A modification.

### 3.6 Circ‐CTNNB1 promotes aerobic glycolysis and OS progression by interaction with RBM15

**FIGURE 6:**
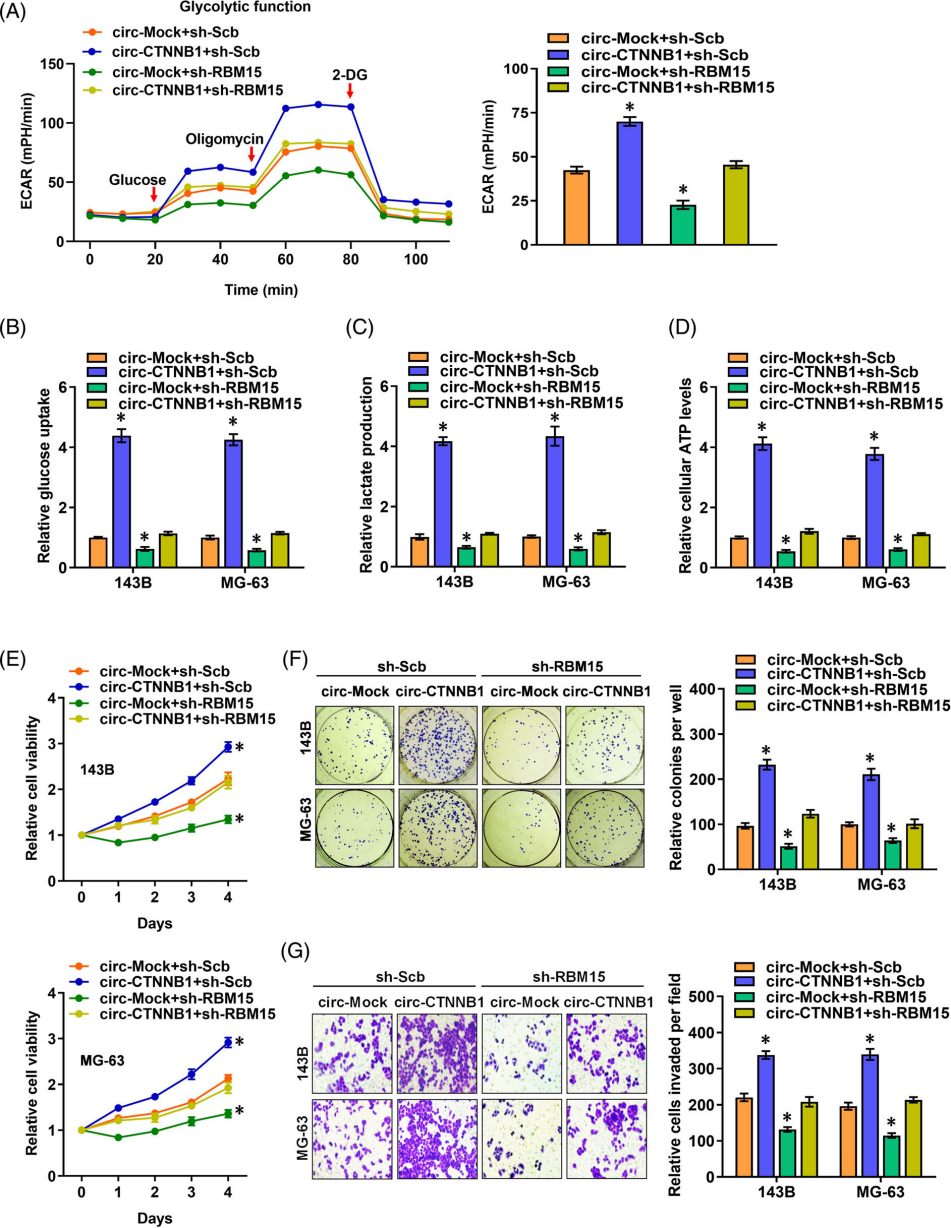
Circ‐CTNNB1 promotes aerobic glycolysis and OS progression by interacting with RBM15. (A) Seahorse tracing curves (left) and ECAR (right) of MG‐63 cells treated with glucose (10 mM), oligomycin (2 μM), or 2‐ deoxyglucose (2‐DG, 50 mM) at indicated points and stably transfected with sh‐Scb or sh‐RBM15 or cotransfected with circ‐Mock or circ‐CTNNB1. (B–D) Glucose uptake (B), lactate production (C), and ATP levels (D) in 143B and MG‐63 cells stably transfected with sh‐Scb or sh‐RBM15 or co‐transfected with circ‐Mock or circ‐CTNNB1. (E) MTT assay showing the vitality of 143B and MG‐63 cells stably transfected with sh‐Scb or sh‐RBM15 or co‐transfected with circ‐Mock or circ‐CTNNB1. (F, G) In vitro growth and invasion of 143B and MG‐63 cells stably transfected with sh‐Scb or sh‐RBM15 or co‐transfected with circ‐Mock or circ‐CTNNB1, as revealed by colony formation (F) and matrigel invasion (G) assay (Data were mean ± SEM of three experiments. ANOVA analysed the difference in A–G. *p<0.05 vs. circ‐Mock+ sh‐Scb). [Correction added on 20 December 2022, after first online publication: the image of matrigel invasion for “circ‐CTNNB1+sh‐Scb” group of MG‐63 cells in Fig. 6G has been corrected]

**FIGURE 7:**
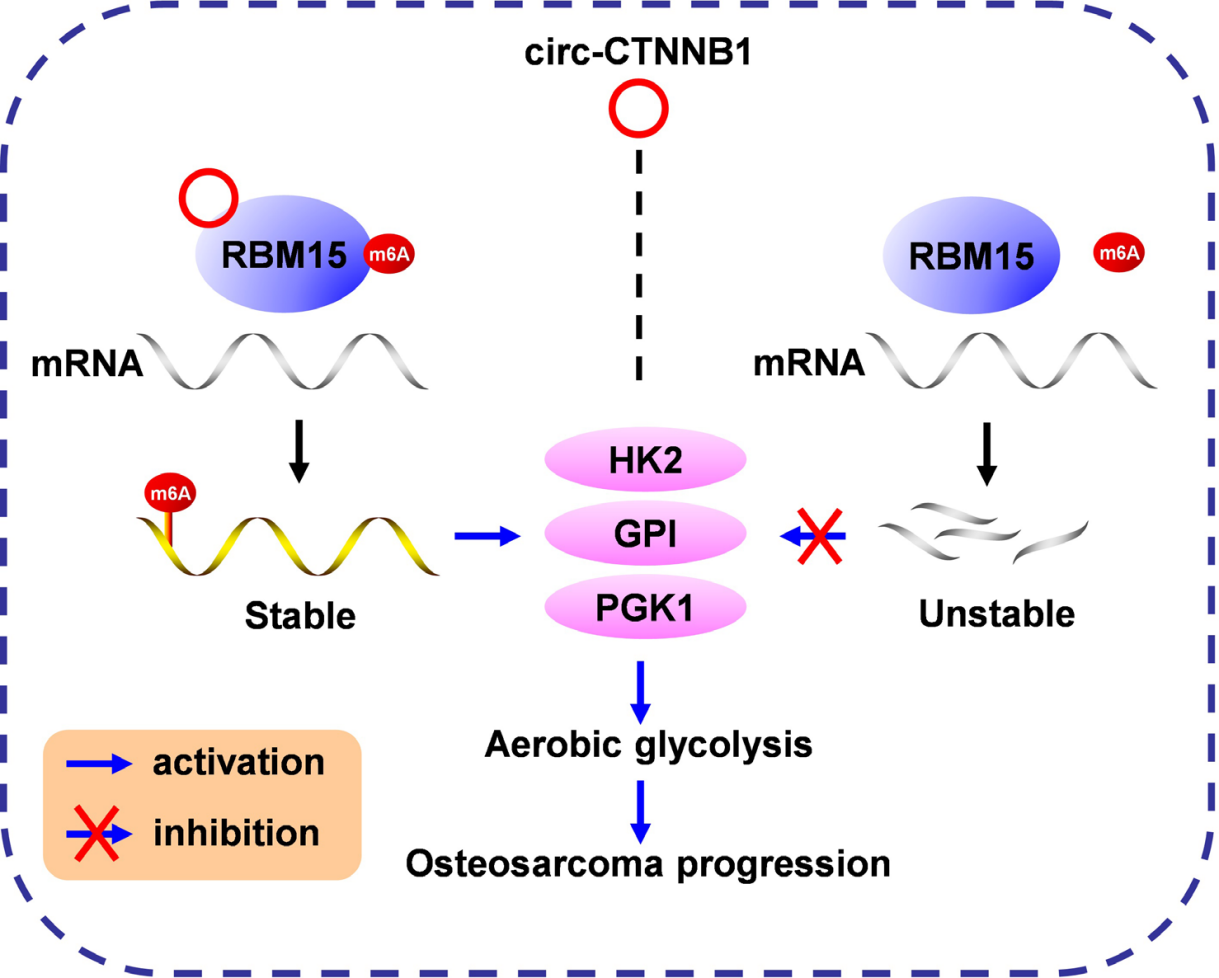
Mechanisms underlying circ‐CTNNB1‐promoted OS progression

Next, the effects of the interplay of circ‐CTNNB1 and RBM15 in OS cells were analysed. The ectopic expression of circ‐CTNNB1 promoted the ECAR and reduced the OCR in OS cells, which were prevented by knockdown of RBM15 (Figure 6A,B). Accordingly, stable interference of RBM15 abolished the increase of the glucose uptake, lactate production and ATP levels induced by circ‐CTNNB1 overexpression in 143B and MG‐63 cells (Figure 6C–E). Notably, stable circ‐CTNNB1 overexpression was facilitated, and MTT, colony formation, and matrigel invasion assays showed the viability, growth and invasiveness of 143B and MG‐63 cells, which were abolished by the knockdown of RBM15, respectively (Figure 6F–H). Taken together, these results showed that circRNA facilitated the aerobic glycolysis process and OS progression through the circ‐CTNNB1/RBM15/m6A axis (Figure 7).

## 4 DISCUSSION

OS is the most prevalent primary bone cancer and ranks as the top malignancy among adolescents.^1^ The 5‐year survival rate of patients with OS has increased in the past 30 years; however, the prognosis in drug‐resistant or metastatic OS is still not satisfactory.^29^ To provide insights into the pathogenesis and metastasis of OS, we identified the novel role of circular RNA circ‐CTNNB1 in regulating OS progression. In this study, we confirmed that upregulated circ‐CTNNB1 exerted an oncogenic role in OS tumour progression in a RBM15‐dependent manner. Mechanistically, circ‐CTNNB1 binds RBM15 to perform the m6A modification of the key aerobic glycolysis genes HK2, GPI and PGK1 and thus stabilize the mRNA levels of the target genes. Consistently, we observed a dynamic aerobic glycolysis process in OS cells (i.e., decrease and increase of the glucose uptake, lactate production, and ATP levels of OS cells for knockdown or overexpression of circ‐CTNNB1, respectively). Then, the process of aerobic glycolysis ensures a survival advantage of tumour cells in the development and progression of cancer.

Recently, numerous studies demonstrate that circRNAs have a crucial role in cancer.^30^ CircRNAs could recruit or sponge proteins to the specific regions of target genes to regulate gene expression.^31^, ^32^ Emerging evidence indicates that circRNAs are aberrant expressed and involved in miRNA inhibition, endothelial–mesenchymal transition (EMT), initiation and progression of OS, and thus could be potential therapeutic targets for OS.^33^, ^34^ For example, circTADA2A promotes OS progression and metastasis via sponging miR‐203a‐3p and facilitating CREB3 expression,^35^ circTCF25 drives carcinogenesis in OS cells by suppressing miR‐206 expression. Our previous study demonstrated the essential roles of the circ‐CTNNB1/DDX3/YY1 axis in cancer progression.^14^ In this study, to identify specific functions and underlying mechanistic involvement of circ‐CTNNB1 in OS, we demonstrate that circ‐CTNNB1 drives aerobic glycolysis and OS progression via m6A modification through interacting with RBM15.

RBM15 is a member of the SPEN (split‐end) family of proteins, which interacts with RNA by binding with spliceosome components,^36^ playing vital roles in the mechanism of mRNA methylation as a m6A methyltransferase ‘writer’,^37^ and exerting oncogenic role in cancer.^38^ RBM15 promoted the invasion, migration and metastasis of OS with a high correlation with metastasis and the decreased survival rate,^39^ and m6A regulators were confirmed to play vital roles in regulating glycolysis of cancer cells.^18^, ^40^ Additionally, studies have revealed that circRNA could regulate aerobic glycolysis and cancer progression in an m6A‐dependent manner.^27^, ^28^ In the current study, gain‐ and loss‐of‐function studies show that RBM15 facilitates aerobic glycolysis and malignant progression of OS cells, suggesting the oncogenic roles of RBM15 in OS. Additionally, our results indicate that RRM1 domain is essential for the interaction between circ‐CTNNB1 and RBM15.

The excessive demand of tumours for nutrients is also associated with severe metabolic challenges. Through metabolic remodelling, tumours have evolved a unique metabolic regulation system.^41^, ^42^ Aerobic glycolysis, which is known as the ‘Warburg effect’,^43^ is the first discovered and most important event in the metabolic reprogramming process. This metabolic reprogramming not only provides ATP for tumour cells but also essential macromolecules for their protein and nucleotide biosynthesis.^44^ The metabolic reprogramming process could be a promising treatment target, and our research provides a potential choice for OS therapy from the metabolic point of view.

The circ‐CTNNB1/RBM15/aerobic glycolysis pathway could be intervened in different ways. CircRNAs are typically knocked down by RNA interference (RNAi)‐based strategies. However, this is accompanied by many limitations, including their instability, lack of cell specificity, low intracellular entry, immune system activation and other off‐target effects.^45^ Using nanoparticles or exosomes as delivery systems can partly improve their efficacy.^46^ Disturbance of the interaction between circ‐CTNNB1 and RBM15 with dominant‐negative mutants or small molecular inhibitors are also relatively specific strategies. In our previous study, the growth and aggressiveness of various other cancer cells were efficiently suppressed by a cell‐penetrating inhibitory peptide, which blocks the interaction of circ‐CTNNB1 and that of the partner protein DDX3.^14^ Therefore, we will further study a way to target this pathway and provide transformation value.

In summary, oncogenic circ‐CTNNB1 is upregulated in OS tissues and cells. High circ‐CTNNB1 expression was associated with increased aggression of OS cells. Circ‐CTNNB1 interacted with RBM15 to facilitate m6A modification of its aerobic glycolysis genes, resulting in more stable mRNA and activation of target genes. Moreover, the aerobic glycolysis level was elevated, which increased the survival advantage of OS cells. Our study provides a potential target for the treatment of OS.

## AUTHOR CONTRIBUTIONS

Feng Yang and Yangyang Liu conceived and performed most of the experiments; Jun Xiao, Bo Li and Jin Zeng accomplished some of in vitro experiments; Yajun Chen and Anpei Hu accomplished in vivo studies; Feng Yang and Anpei Hu undertook the mining of publicly available datasets; Zhili Liu critically reviewed the manuscript; Feng Yang and Hucheng Liu wrote the manuscript. All authors read and approved the final manuscript.

## CONFLICT OF INTEREST

The authors declare that they have no competing interests.

## Supporting information


**Table S1.** Primer sets used for qPCR, RT‐PCR, and RIP
**Table S2**. Oligonucleotide sets used for constructs and short hairpin RNAs
**Table S3**. Screening for proteins and target genes (Figure 3A and 4F)
**Figure S1**. Expression profiles of circ‐CTNNB1. (A) Real‐time qRT‐PCR indicating the distribution of GAPDH, U1, and circ‐CTNNB1 in the cytoplasm and nuclear fractions of 143B and MG‐63 cells. (B) Real‐time qRT‐PCR analysis verified the effective overexpression in 143B and MG‐63 cells stably transfected with circ‐Mock, circ‐CTNNB1, sh‐Scb, or sh‐circ‐CTNNB1 #1, #2. (C) Real‐time qRT‐PCR assay indicating the levels of CTNNB1 in 143B and MG‐63 cells with overexpression or knockdown of circ‐CTNNB1. (Data were mean ± SEM of three experiments. Student's *t*‐test and ANOVA analysed the difference in B, C. *P < 0.05 vs. circ‐Mock, sh‐Scb).
**Figure S2**. circ‐CTNNB1 promotes aerobic glycolysis in OS. (A, B) Seahorse tracing curves (A) ECAR and OCR (B) of 143B cells stably transfected with circ‐Mock and circ‐CTNNB1, and those treated with glucose (10 mM), oligomycin (2 μM), or 2‐deoxyglucose (2‐DG, 50 mM) at indicated points. (C–E) The glucose uptake (C), lactate production (D), and ATP levels (E) in 143B and MG‐63 cells stably transfected with circ‐Mock, circ‐CTNNB1 and those treated with 2‐DG (10 mM) for 48 h. Representative images (left panel) and quantification (right panel) of colony formation (F) and matrigel invasion (G) assay indicating the growth and invasion of 143B cells stably transfected with mock or circ‐CTNNB1 and those treated with 2‐DG (10 mM) for 48 h. (H) In vivo imaging (left panel), counts of lung metastasis (right panel), and immunohistochemical staining of Ki‐67 and CD31 within lung metastasis tumours of nude mice treated with tail vein injection of 143B cells stably transfected with circ‐Mock or circ‐CTNNB1 and those treated with daily oral gavage of 2‐DG (1 g·kg − 1, *n* = 5 for each group). (Data were mean ± SEM of three experiments. Student's *t*‐test and ANOVA analysed the difference in A–H. *p < 0.05 vs. circ‐Mock).
**Figure S3**. RBM15 is overexpressed in OS. (A) Relative RBM15 levels in OS compared with normal tissues in GSE87624. (B, C) Western blot (B, *n* = 5) and Real‐time qRT‐PCR assay (C, *n* = 20) showing the relative levels of RBM15 in the adjacent normal tissue (N) and tumour tissues (T) of OS. (D, E) Western blot assay (D) and real‐time qRT‐PCR (E) assay revealing the mRNA and protein levels of RBM15 in 143B and MG‐63 cells stably transfected with circ‐Mock, circ‐CTNNB1, sh‐Scb, or sh‐circ‐CTNNB1 #1, #2. (Data were mean ± SEM of three experiments. Student's *t*‐test and ANOVA compared the difference in A, B, D. *p < 0.05, **p < 0.01 vs. circ‐Mock or sh‐Scb).
**Figure S4**. RBM15 promotes aerobic glycolysis in OS. (A, B) Seahorse tracing curves (A), ECAR and OCR (B) of 143B cells stably transfected with Mock and RBM15, and those treated with glucose (10 mM), oligomycin (2 μM), or 2‐deoxyglucose (2‐DG, 50 mM) at indicated points. (C–E) The glucose uptake (C), lactate production (D), and ATP levels (E) in 143B and MG‐63 cells stably transfected with Mock, RBM15. (Data were mean ± SEM of three experiments. Student's *t*‐test and ANOVA analysed the difference in A–E. *p < 0.05 vs. Mock).
**Figure S5**. circ‐CTNNB1 facilitates RBM15‐mediated gene activation. (A, B) Real‐time qRT‐PCR (A) and western blot (B) assay indicating the transcript and protein expression levels of GPI, HK2 and PGK1 mRNA (normalized to β‐actin) in MG‐63 cells stably transfected with sh‐Scb or sh‐RBM15, and those cotransfected with circ‐Mock or circ‐CTNNB1. (C, D) Dual‐luciferase assay revealing the promoter activity of GPI, HK2, and PGK1 in 143B (C) and MG‐63 (D) cells stably transfected with sh‐Scb or sh‐RBM15, and those cotransfected with circ‐Mock or circ‐CTNNB1. (E–G) The mRNA half‐life (E), transcript (F) and m6A levels (G) of GPI, HK2, and PGK1 in 143B cells stably transfected with sh‐Scb or sh‐circ‐CTNNB1 or co‐transfected with RBM15 WT or RBM15 ΔRRM1. (Data were mean ± SEM of three experiments. Student's *t*‐test and ANOVA analysed the difference in A, C–G. *p < 0.05).

## Data Availability

The data supporting the conclusions of this article are included in this article and its additional files.
