# Supplementary material for: Circ‐CTNNB1 drives aerobic glycolysis and osteosarcoma progression via m6A modification through interacting with RBM15
Source: Cell Prolif. 2022 Oct 1;56(1):e13344. doi: 10.1111/cpr.13344 (PMC9816931; doi:10.1111/cpr.13344)
Supplement: Supplementary file 1 — Table S1. Primer sets used for qPCR, RT‐PCR, and RIP Table S2. Oligonucleotide sets used for constructs and short hairpin RNAs Table S3. Screening for proteins and target genes (Figure 3A and 4F) Figure S1. Expression profiles of circ‐CTNNB1. (A) Real‐time qRT‐PCR indicating the distribution of GAPDH, U1, and circ‐CTNNB1 in the cytoplasm and nuclear fractions of 143B and MG‐63 cells. (B) Real‐time qRT‐PCR analysis verified the effective overexpression in 143B and MG‐63 cells stably transfected with circ‐Mock, circ‐CTNNB1, sh‐Scb, or sh‐circ‐CTNNB1 #1, #2. (C) Real‐time qRT‐PCR assay indicating the levels of CTNNB1 in 143B and MG‐63 cells with overexpression or knockdown of circ‐CTNNB1. (Data were mean ± SEM of three experiments. Student's t‐test and ANOVA analysed the difference in B, C. *P < 0.05 vs. circ‐Mock, sh‐Scb). Figure S2. circ‐CTNNB1 promotes aerobic glycolysis in OS. (A, B) Seahorse tracing curves (A) ECAR and OCR (B) of 143B cells stably transfected with circ‐Mock and circ‐CTNNB1, and those treated with glucose (10 mM), oligomycin (2 μM), or 2‐deoxyglucose (2‐DG, 50 mM) at indicated points. (C–E) The glucose uptake (C), lactate production (D), and ATP levels (E) in 143B and MG‐63 cells stably transfected with circ‐Mock, circ‐CTNNB1 and those treated with 2‐DG (10 mM) for 48 h. Representative images (left panel) and quantification (right panel) of colony formation (F) and matrigel invasion (G) assay indicating the growth and invasion of 143B cells stably transfected with mock or circ‐CTNNB1 and those treated with 2‐DG (10 mM) for 48 h. (H) In vivo imaging (left panel), counts of lung metastasis (right panel), and immunohistochemical staining of Ki‐67 and CD31 within lung metastasis tumours of nude mice treated with tail vein injection of 143B cells stably transfected with circ‐Mock or circ‐CTNNB1 and those treated with daily oral gavage of 2‐DG (1 g·kg − 1, n = 5 for each group). (Data were mean ± SEM of three experiments. Student's t‐test and ANOVA analy [file CPR-56-e13344-s001.docx]

**Additional file1：Table**

**Table S1 Primer sets used for qPCR, RT-PCR, and RIP**

| **Primer set 1** | **Primers** | **Sequence** | **Product size (bp)** |
| --- | --- | --- | --- |
| ACTB | Forward | 5'-TGCCCATCTACGAGGGGTATG-3' | 156 |
| (Convergent) | Reverse | 5'-TCTCCTTAATGTCACGCACGATTT-3' |  |
| ACTB | Forward | 5'-AAATCGTGCGTGACATTAAGGAGA-3’ | - |
| (Divergent) | Reverse | 5'-CATACCCCTCGTAGATGGGCA-3’ |  |
| circ-CTNNB1 | Forward | 5'-TTTTCTTTTACATGCCCCCTCT-3’ | 305 |
| (Convergent) | Reverse | 5'-GGAAGAAGAGAGTTTTTGTGTCCTT-3’ |  |
| circ-CTNNB1 | Forward | 5'-AAGGACACAAAAACTCTCTTCTTCC-3’ | 120 |
| (Divergent) | Reverse | 5'-AGAGGGGGCATGTAAAAGAAAA-3’ |  |
| CTNNB1 | Forward | 5'-CGGGCTGGTGACAGGGAAGAC-3’ | 209 |
|  | Reverse | 5'-GCGGGACAAAGGGCAAGATTT-3’ |  |
| ALDOA | Forward | 5'-GTGGGCATCAAGGTAGACAA-3’ | 226 |
|  | Reverse | 5'-GCTGGCAGATACTGGCATAA-3’ |  |
| ENO1 | Forward | 5'-GATGACTGGGGAGCTTGGCAGAAG-3’ | 134 |
|  | Reverse | 5'-TTGAGCAGGAGGCAGTTGCAGGAC-3’ |  |
| GPI | Forward | 5'-AGTTCTGGGATTGGGTGGGA-3' | 230 |
|  | Reverse | 5'-ATAGGGCAGCATGGCGTGTG-3' |  |
| HK2 | Forward | 5'-TGGAGCGAGGTCTGAGCAAG-3' | 145 |
|  | Reverse | 5'-ACCAGCAGGACCCGGAAATT-3' |  |
| PGK1 | Forward | 5'-AGCCAAGATTGTCAAAGACCT-3' | 255 |
|  | Reverse | 5'-GCTTCCCATTCAAATACCCC-3' |  |
| GPI (3’-UTR) | Forward | 5'-GTTAGCCAGGATGGTCTTGATC-3' | 303 |
|  | Reverse | 5'-TACCCTTTAGAGGTTTCCTATTTG-3' |  |
| HK2 (3’-UTR) | Forward | 5'-GGTTGCTTCTGGCTCCTCCTTC-3' | 261 |
|  | Reverse | 5'-TTGGTGACTTCCTTTATCTTGT-3' |  |
| PGK1 (3’-UTR) | Forward | 5'-TTCCTACTGATTACCTTTCCTCCAA-3' | 453 |
|  | Reverse | 5'-ACAGGTGATCTGCCCGCCTTG-3' |  |
|  |  |  |  |

ACTB, beta-actin; circ-CTNNB1, circular RNA CTNNB1; U1, U1 small nuclear 1; GAPDH, glyceraldehyde 3-phosphate dehydrogenase; ALDOA, aldolase, fructose-bisphosphate A; ENO1, enolase 1; GPI, glucose-6-phosphate isomerase; HK2, hexokinase 2; PGK1, phosphoglycerate kinase 1; 3’-UTR, 3’-Untranslated regions; RIP, RNA immunoprecipitation.

**Table S2 Oligonucleotide sets used for constructs and short hairpin RNAs**

| **Oligo Set** | **Sequences** |
| --- | --- |
| pLCDH-circ-CTNNB1 | 5'-CCGGAATTCTGAAATATGCTATCTTACAGGTATATAAAAAGAGAAGATGAGA-3' (sense); |
|  | 5'-CGCGGATCCTCAAGAAAAAATATATTCACCTTTTATTTTAAATCTCTTTTCT-3' (antisense) |
| sh-Scb | 5'-AGGGATACAAGCATATACCACTCGAGTGGTATATGCTTGTATCCCTC-3' (sense); |
|  | 5'-GAGGGATACAAGCATATACCACTCGAGTGGTATATGCTTGTATCCCT-3' (antisense) |
| sh-circ-CTNNB1 #1 | 5'-CCGGTTAAAATAAAAGGTATATAAAACTCGAGTTTTATATACCTTTTATTTTATTTTTG-3' (sense); |
|  | 5’-GATCCAAAAATAAAATAAAAGGTATATAAAACTCGAGTTTTATATACCTTTTATTTTAA-3’ (antisense) |
| sh-circ-CTNNB1 #2 | 5'-CCGGTAGATTTAAAATAAAAGGTATACTCGAGTATACCTTTTATTTTAAATCTTTTTTG-3' (sense); |
|  | 5'-GATCCAAAAAAGATTTAAAATAAAAGGTATACTCGAGTATACCTTTTATTTTAAATCTA-3' (antisense) |
| sh-RBM15 #1 | 5’-CCGGTAAGACTCTGAAGATAAGCGAGCTCGAGCTCGCTTATCTTCAGAGTCTTTTTTTG-3 (sense); |
|  | 5’-GATCCAAAAAAAGACTCTGAAGATAAGCGAGCTCGAGCTCGCTTATCTTCAGAGTCTTA-3’ (antisense) |
| sh-RBM15 #2 | 5’-CCGGTAACCTAGACATCACTGTAACGCTCGAGCGTTACAGTGATGTCTAGGTTTTTTTG-3’ (sense); |
|  | 5’-GATCCAAAAAAACCTAGACATCACTGTAACGCTCGAGCGTTACAGTGATGTCTAGGTTA-3’ (antisense) |
| pCMV-3Tag-1A-RBM15 Full | 5’-CGCGGATCCATGAGGACTGCGGGGCGGGACC-3’ (sense) |
|  | 5’-CCGGAATTCCTATAACAGGGTCAGCGCCAAG-3’ (antisense) |
| pCMV-3Tag-1A- RBM15-ΔRRM1 | 5’-CGCGGATCCCCTTTAGACAAAGATACTTATC-3’ (sense) |
|  | 5’-CCGGAATTCCTATAACAGGGTCAGCGCCAAG-3’ (antisense) |
| pCMV-3Tag-1A- RBM15-ΔSPOC | 5’-CGCGGATCCATGAGGACTGCGGGGCGGGACC-3’ (sense) |
|  | 5’-CCGGAATTCAGGCTGCAGATACTGCTGCTGG-3’ (antisense) |
| pCMV-3Tag-1A- RBM15-RRM1+2 | 5’-CGCGGATCCATGAGGACTGCGGGGCGGGACC-3’ (sense) |
|  | 5’-CCGGAATTCGGGTGTAGCTTTACCATAACCA-3’ (antisense) |
| pCMV-3Tag-1A- RBM15-RRM1 | 5’-CGCGGATCCATGAGGACTGCGGGGCGGGACC-3’ (sense) |
|  | 5’-CCGGAATTCAGGGGAGCGGCTGCGGCGCCGG-3’ (antisense) |
| pGL3-GPI (-1854/+247) | 5’-CGGGGTACCTCTCTGATGGATTCAGGCAGGGGTA-3’ (sense); |
|  | 5’-GCCGCTCGAGGAAGTGGCAGTGGGGAGGGTGGGCA-3’(antisense) |
| pGL3-HK2 (-1813/+424) | 5’-CGGGGTACCCAAGGAAAGCCTGATGAGGTAGAAG-3’ (sense) |
|  | 5’-GCCGCTCGAGTGATGATGTGAATGAACTGGGTAGA-3’ (antisense) |
| pGL3-PGK1 (-882/+246) | 5’-CGGGGTACCCTAAGAACTTGGACACCCTCCACG-3’ (sense) |
|  | 5’-GCCGCTCGAGCAGAATTACCTCATAACGACCCGC-3’ (antisense) |
| psiCHECK2-GPI 3’-UTR | 5’-GCCGCTCGAGTTTCCTGTGATGGTGCTTTATG-3’ (sense) |
|  | 5’-ATTTGCGGCCGCTACCCTTTAGAGGTTTCCTATTTG-3’ (antisense) |
|  |  |
| psiCHECK2-HK2 3’-UTR | 5’-GCCGCTCGAGCCCCTGAAATCGGAAGGGACT-3’ (sense) |
|  | 5’-ATTTGCGGCCGCATCCAGGTTTAATGTCTGTGCTTGT-3’ (antisense) |
| psiCHECK2-PGK1 3’-UTR | 5’-GCCGCTCGAGCTGAGAAAGGGTGCCTGGGAGG-3’ (sense) |
|  | 5’-ATTTGCGGCCGCCCCAAGCAGGAATGCAGTAGCG-3’ (antisense) |
| psiCHECK2-GPI 3’-UTR-Mut | 5’-TCACTTCAGCCTCTGCCTGGTGGCAGGCCAAGTCTTTATTTACATAGG-3’ (sense) |
|  | 5’-TTGGCCTGCCACCAGGCAGAGGCTGAAGTGAATGCTCCTTTGTCTCCA-3’ (antisense) |
| psiCHECK2-HK2 3’-UTR-Mut | 5’-CCAGAGATGGAGGCCGAGCCAATAGCCTGAAGAGACCACAGCAAT-3’ (sense) |
|  | 5’-GCTATTGGCTCGGCCTCCATCTCTGGCCCTGCCTTCCACACACTTC-3’ (antisense) |
| psiCHECK2-PGK1 3’-UTR Mut | 5’-GCCTGTAATCCCAGGCCTTTGGGAGGCCAAGGCGGGCAGATCACCTGT -3’ (sense) |
|  | 5’-CTTGGCCTCCCAAAGGCCTGGGATTACAGGCGTAAGCCACCATGCC-3’ (antisense) |
|  |  |

ALDOA, aldolase, fructose-bisphosphate A; ENO1, enolase 1; GPI, glucose-6-phosphate isomerase; HK2, hexokinase 2; PGK1, phosphoglycerate kinase 1; 3’-UTR, 3’-Untranslated regions; RBM15, RNA binding motif protein 15.

**Table S3 screening for proteins and target genes (Fig. 3A)**

**RNA pull down-MS m6A regulators DEGs in GSE87624 Overlap**

RBM15

IGF2BP1

1924 genes

(not shown)

METTL3

METTL14

METTL5

METTL16

TRMT112

ZCCHC4

HAKAI

WTAP

VIRMA

RBM15

RBM15B

ZC3H13

FTO

ALKBH5

YTHDF1

YTHDF2

YTHDF3

YTHDC1

YTHDC2

HNRNPC

HNRNPG

HNRNPA2B1

IGF2BP1

IGF2BP2

IGF2BP3

EIF3

PRRC2A

SND1

ACTBL2 KRT17

ACTN1 KRT18

ACTN4 KRT2

ACTR2 KRT7

ACTR3 KRT8

AHNAK KRT9

ALPI LIMA1

ALPL LMNA

ANXA2 LMO7

ARPC1B MISP

ATP5A1 MYH14

ATP5B MYH9

ATP6V1A MYL6

BASP1 MYO1C

CAPZA2 MYO1E

CAPZB MYO6

CORO1C MYOF

CSTA NDUFS1

CTTN NEXN

DBN1 NPM1

DSP PDIA3

DYDC1 PLEC

POTEJ EEF1A1P5

EEF2 PPP1R12A

EPPK1 PPP1R9B

FLII PRDX1

FLNA RBM15

FOLR1 RBMX

FSCN1 SCIN

GNB2 SDHA

GSN SDHB

SVIL HIST1H2BN

SYNPO HIST1H4A

TFRC HNRNPC

TJP1 HNRNPH1

TMOD3 HSP90AB1

HSPA5 TUBA1B

HSPA8 TUBB4B

HSPA9 TUFM

HSPD1 ULBP1

IGF2BP1 UQCRC1

ITGB1 UQCRC2

JUP VIL1

KRT1 VIM

KRT10

MS: Mass spectrometry; DEGs: Differentially expressed genes

**Table S3 screening for proteins and target genes (Fig. 4F)**

**Glycolysis gene RBM15 CLiP-seq Overlap Key regulators in glycolysis**

ALDOA

ENO1

GPI

HK2

PGK1

PGM1

ENO3

LDHA

HK1

PFKM

ACSS1

PDHB

ALDH9A1

TPI1

PCK2

PFKL

PGAM1

ENO2

AKR1A1

GPI

ALDH1B1

GALM

ALDOA

HK2

PFKP

ADH5

MINPP1

G6PC

ALDH7A1

PDHA1

ALDH3A2

LDHB

BPGM

ENO1

ALDH1A3

ADPGK

GAPDH

PGK1

PCK1

ACSS2

PGM2

DLD

PKM

G6PC3

ACSS1 GALM

ACSS2 GAPDH

ADH1A GAPDHS

ADH1B GCK

ADH1C GPI

ADH4 HK1

ADH5 HK2

ADH6 HK3

ADH7 HKDC1

ADPGK LDHA

AKR1A1 LDHAL6A

ALDH1A3 LDHAL6B

ALDH1B1 LDHB

ALDH2 LDHC

ALDH3A1 MINPP1

ALDH3A2 PCK1

ALDH3B1 PCK2

ALDH3B2 PDHA1

ALDH7A1 PDHA2

ALDH9A1 PDHB

ALDOA PFKL

ALDOB PFKM

ALDOC PFKP

BPGM PGAM1

DLAT PGAM2

DLD PGAM4

ENO1 PGK1

ENO2 PGK2

ENO3 PGM1

FBP1 PGM2

FBP2 PKLR

G6PC PKM

G6PC2 TPI1

G6PC3

13125 genes

(not shown)

**Additional file 2：Figure**


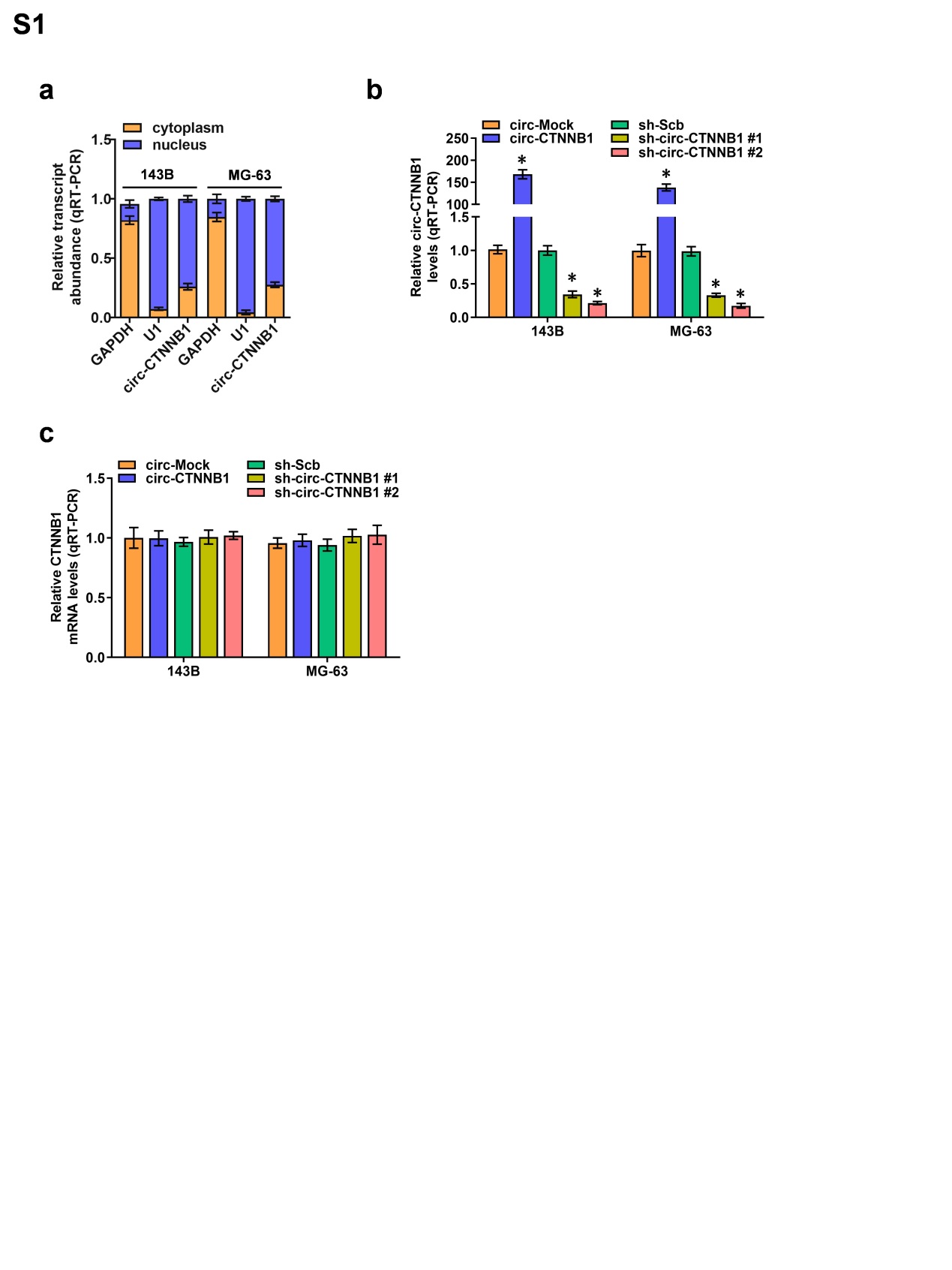


**Fig. S1 Expression profiles of circ-CTNNB1.** **a** Real-time qRT-PCR indicating the distribution of GAPDH, U1, and circ-CTNNB1 in the cytoplasm and nuclear fractions of 143B and MG-63 cells. **b** Real-time qRT-PCR analysis verified the effective overexpression in 143B and MG-63 cells stably transfected with circ-Mock, circ-CTNNB1, sh-Scb, or sh-circ-CTNNB1 #1, #2. **c** Real-time qRT-PCR assay indicating the levels of CTNNB1 in 143B and MG-63 cells with overexpression or knockdown of circ-CTNNB1. (Data were mean ± SEM of three experiments. Student’s *t* test and ANOVA analyzed the difference in b, c. **P*<0.05 vs. circ-Mock, sh-Scb).

**
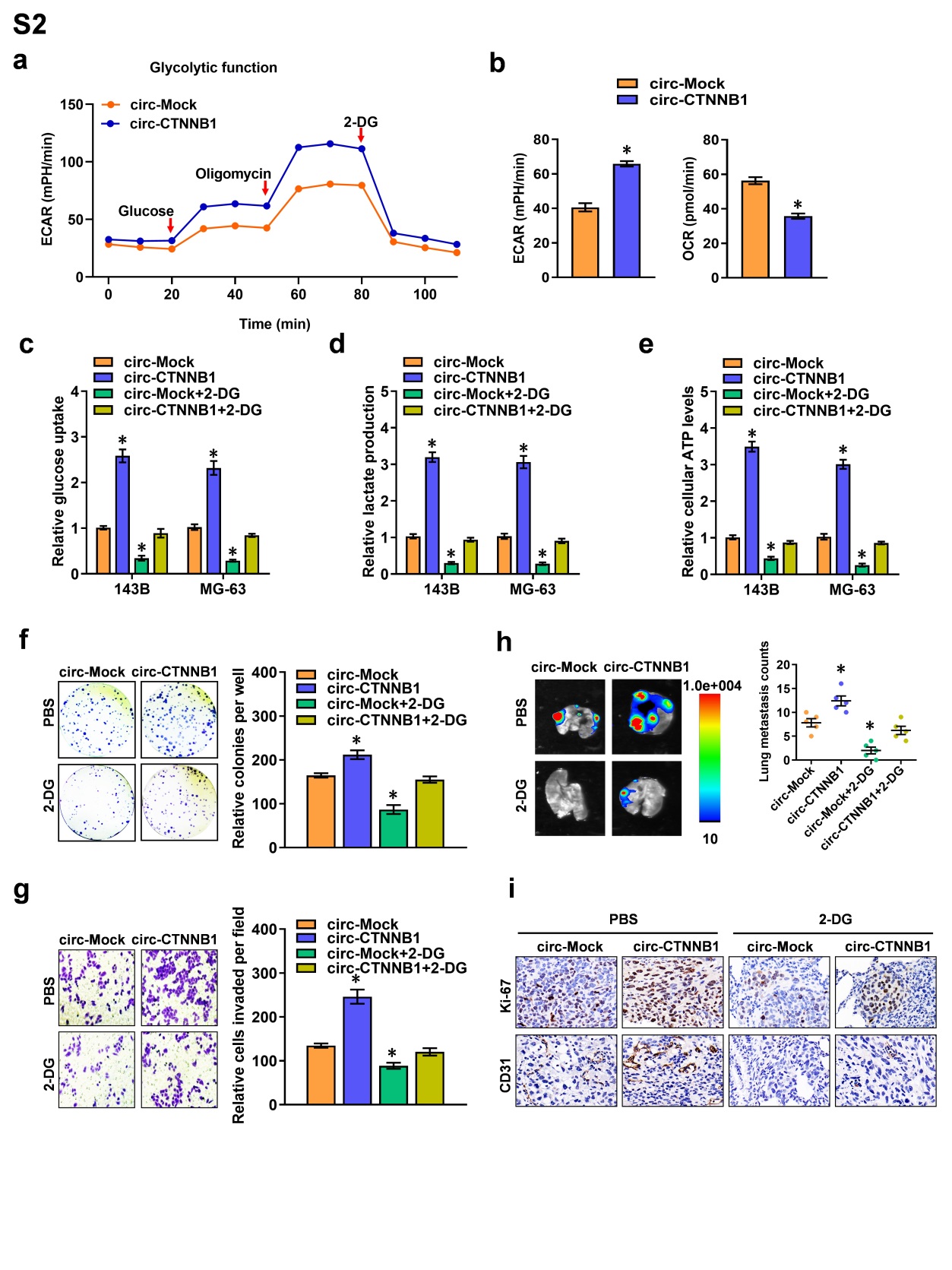
Fig. S2** **circ-CTNNB1 promotes aerobic glycolysis in OS. a & b** Seahorse tracing curves (**a**) ECAR and OCR (**b**) of 143B cells stably transfected with circ-Mock and circ-CTNNB1, and those treated with glucose (10 mM), oligomycin (2 μM), or 2-deoxyglucose (2-DG, 50 mM) at indicated points. c-e The glucose uptake (**c**), lactate production (**d**), and ATP levels (**e**) in 143B and MG-63 cells stably transfected with circ-Mock, circ-CTNNB1 and those treated with 2-DG (10 mM) for 48 hrs. Representative images (left panel) and quantification (right panel) of colony formation (**f**) and matrigel invasion (**g**) assay indicating the growth and invasion of 143B cells stably transfected with mock or circ-CTNNB1 and those treated with 2-DG (10 mM) for 48 h. (**h**) In vivo imaging (left panel), counts of lung metastasis (right panel), and immunohistochemical staining of Ki-67 and CD31 within lung metastasis tumors of nude mice treated with tail vein injection of 143B cells stably transfected with circ-Mock or circ-CTNNB1 and those treated with daily oral gavage of 2-DG (1 g⋅kg−1, n=5 for each group). (Data were mean ± SEM of three experiments. Student’s t test and ANOVA analyzed the difference in a-h. *P<0.05 vs. circ-Mock).


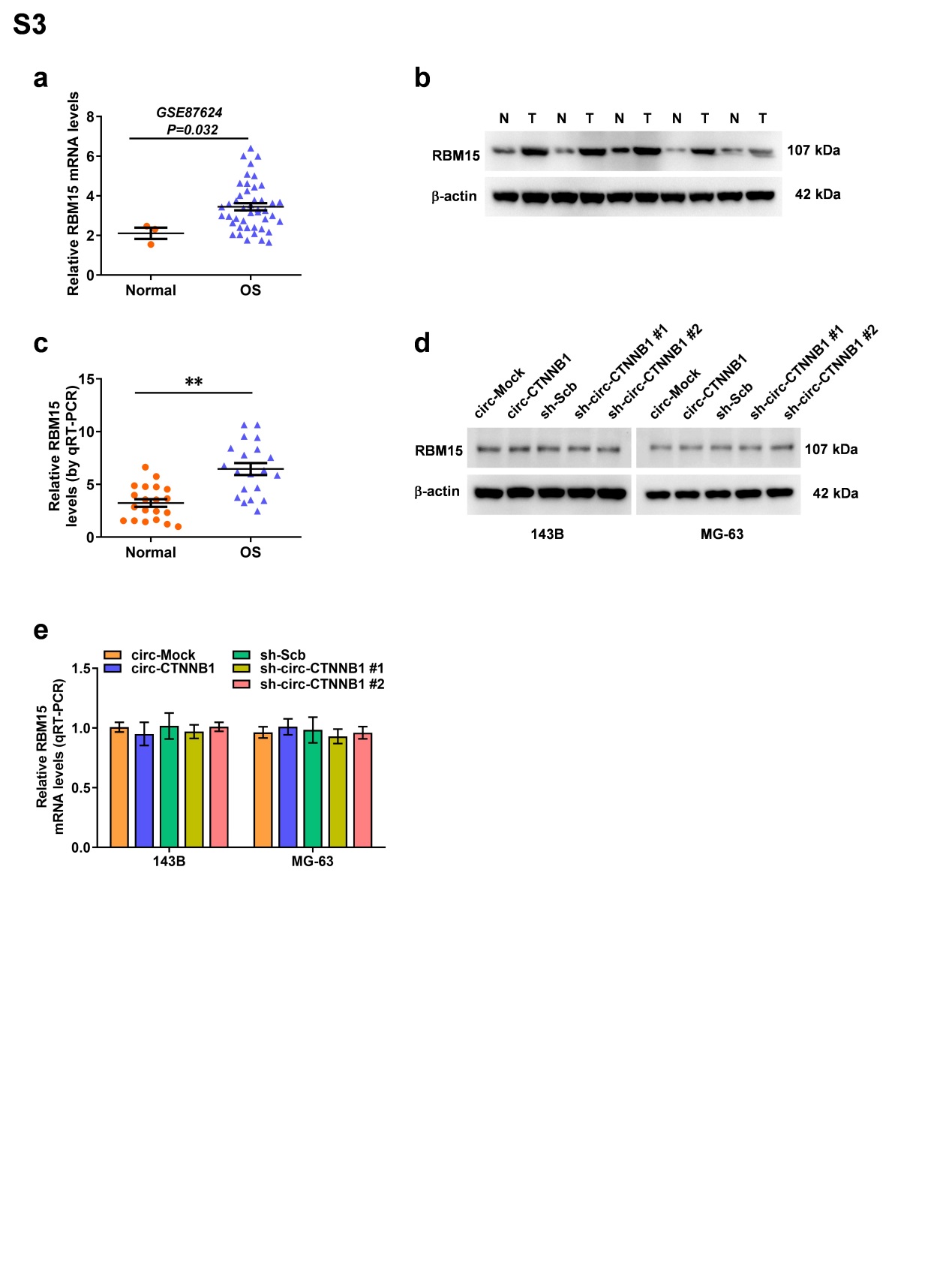


**Fig. S3 RBM15 is overexpressed in OS. a** Relative RBM15 levels in OS compared with normal tissues in GSE87624. **b** & **c** Western blot (**b,** n=5) and Real-time qRT-PCR assay (**c,** n=20) showing the relative levels of RBM15 in the adjacent normal tissue (N) and tumor tissues (T) of OS**. d** & **e** Western blot assay (**d**) and Real-time qRT-PCR (**e**) assay revealing the mRNA and protein levels of RBM15 in 143B and MG-63 cells stably transfected with circ-Mock, circ-CTNNB1, sh-Scb, or sh-circ-CTNNB1 #1, #2. (Data were mean ± SEM of three experiments. Student’s *t* test and ANOVA compared the difference in **a, b, d**. **P*<0.05, ***P*<0.01 vs. circ-Mock or sh-Scb).


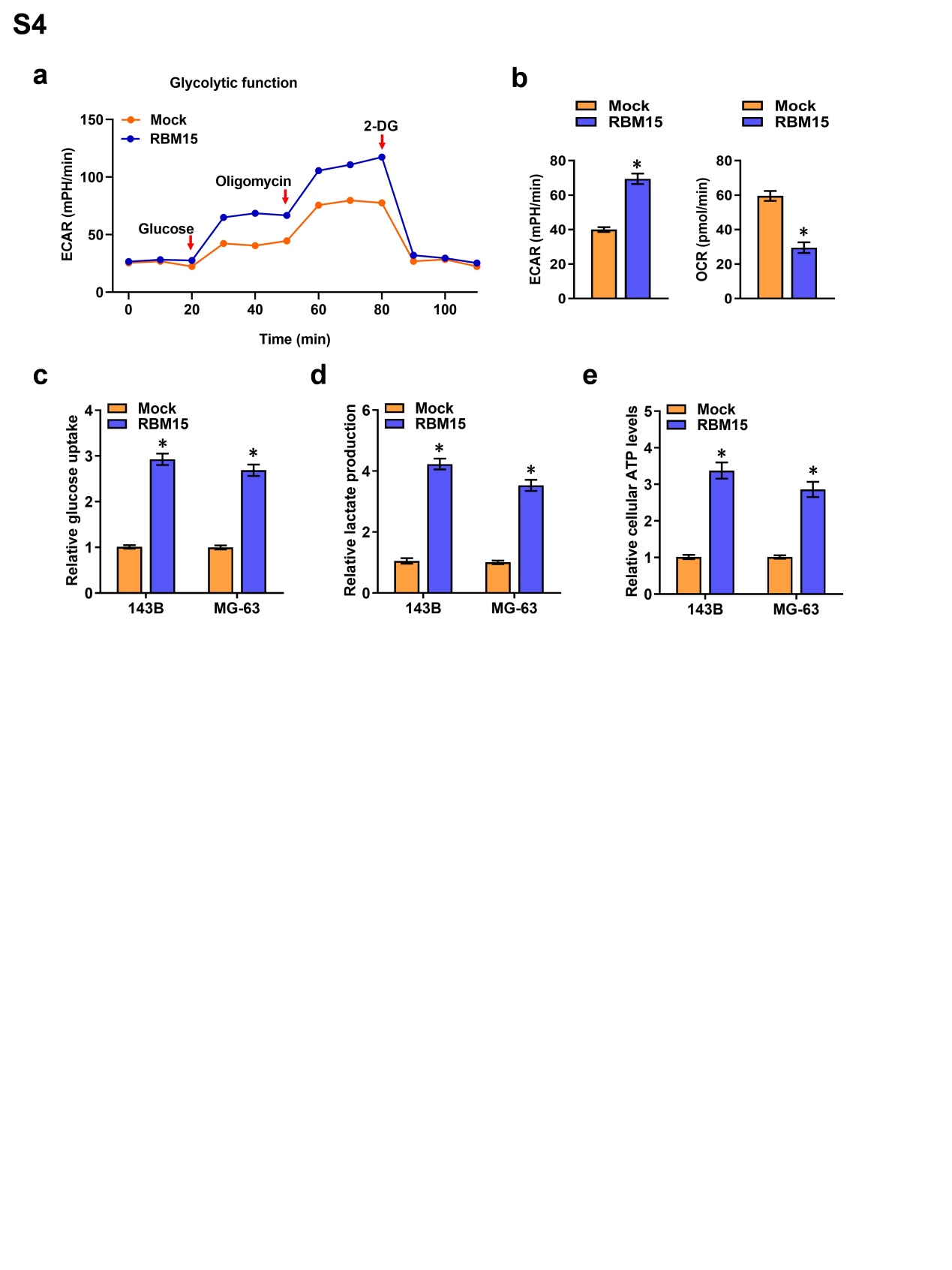


**Fig. S4 RBM15 promotes aerobic glycolysis in OS. a** & **b** Seahorse tracing curves (**a**), ECAR and OCR (**b**) of 143B cells stably transfected with Mock and RBM15, and those treated with glucose (10 mM), oligomycin (2 μM), or 2-deoxyglucose (2-DG, 50 mM) at indicated points. **c-e** The glucose uptake (**c**), lactate production (**d**), and ATP levels (**e**) in 143B and MG-63 cells stably transfected with Mock, RBM15. (Data were mean ± SEM of three experiments. Student’s *t* test and ANOVA analyzed the difference in a-e. **P*<0.05 vs. Mock).


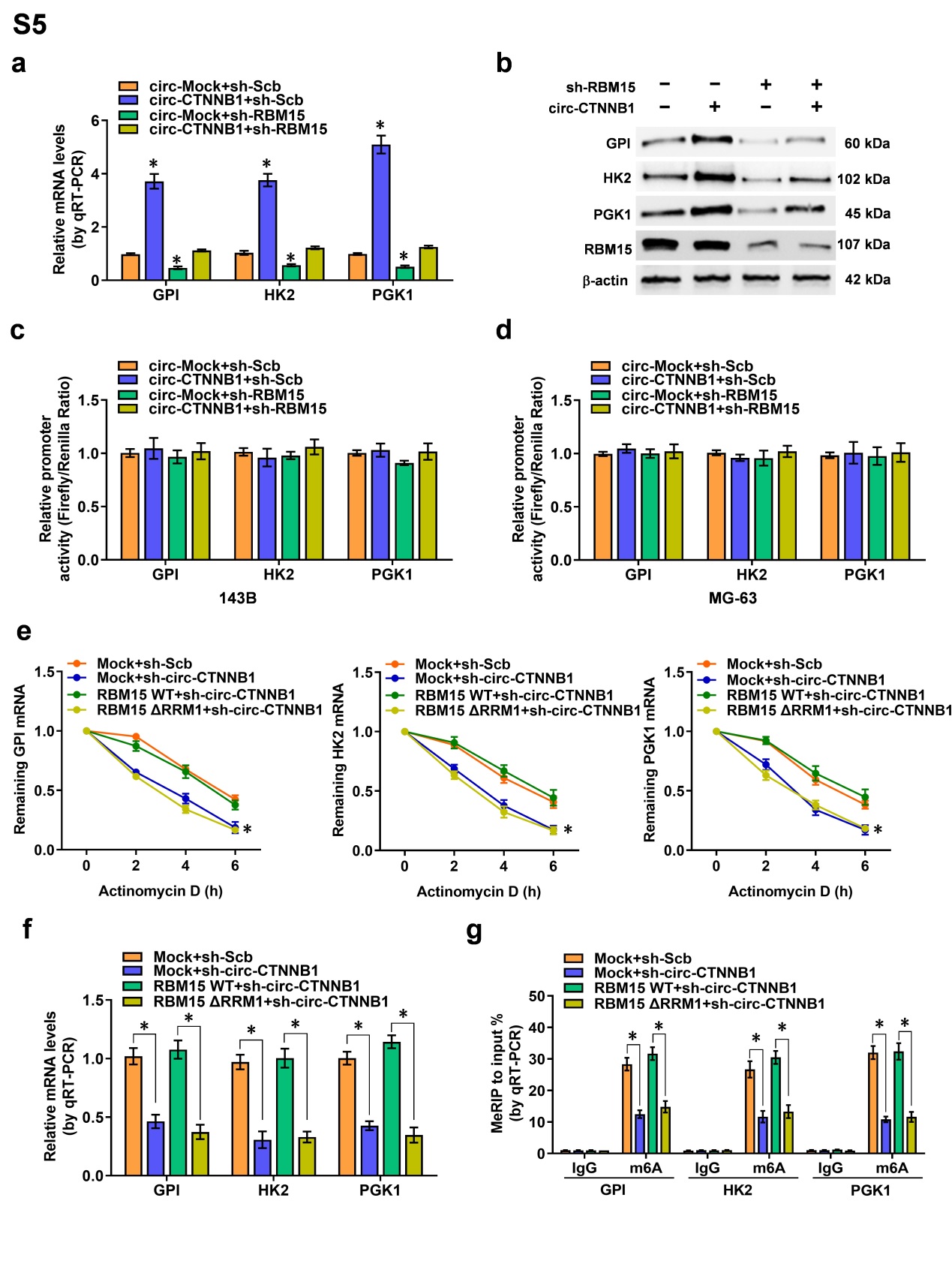
**Fig. S5 circ-CTNNB1 facilitates RBM15-mediated gene activation. a** & **b** Real-time qRT-PCR (**a**) and western blot (**b**) assay indicating the transcript and protein expression levels of GPI, HK2 and PGK1 mRNA (normalized to β-actin) in MG-63 cells stably transfected with sh-Scb or sh-RBM15, and those cotransfected with circ-Mock or circ-CTNNB1. **c** & **d** Dual-luciferase assay revealing the promoter activity of GPI, HK2 and PGK1 in 143B (**c**) and MG-63 (**d**) cells stably transfected with sh-Scb or sh-RBM15, and those cotransfected with circ-Mock or circ-CTNNB1.

**e-g** the mRNA half-life(**e**), transcript (**f**) and m6A levels (**g**) of GPI, HK2, and PGK1 in 143B cells stably transfected with sh-Scb or sh-circ-CTNNB1 or co-transfected with RBM15 WT or RBM15 ΔRRM1. (Data were mean ± SEM of three experiments. Student’s *t* test and ANOVA analyzed the difference in a, c-g. **P*<0.05).
